# Aging clocks delineate neuron types vulnerable or resilient to neurodegeneration and identify neuroprotective interventions

**DOI:** 10.1038/s43587-026-01067-5

**Published:** 2026-02-03

**Authors:** Christian Gallrein, David H. Meyer, Yvonne Woitzat, Valeria Ramirez-Ramirez, Thanh Vuong-Brender, Janine Kirstein, Björn Schumacher

**Affiliations:** 1 Institute for Genome Stability in Aging and Disease, Medical Faculty, University and University Hospital of Cologne, Cologne, Germany; 2 Cologne Excellence Cluster for Cellular Stress Responses in Aging-Associated Diseases (CECAD), University of Cologne, Cologne, Germany; 3 Leibniz Institute on Aging – Fritz-Lipmann-Institute (FLI), Jena, Germany; 4 Friedrich Schiller University, Institute for Biochemistry and Biophysics, Jena, Germany

**Keywords:** Neural ageing, Ageing, Data integration, Ageing

## Abstract

Different neuron types show distinct susceptibility to age-dependent degeneration, yet the underlying mechanisms are poorly understood. Here we applied aging clocks to single neuron types in *Caenorhabditis* *elegans* and found that distinct neurons differ in their biological age. Ciliated sensory neurons with high neuropeptide and protein biosynthesis gene expression show accelerated aging and degeneration, correlating with loss of function, which could be prevented by pharmacological inhibition of translation. We show that the *C. elegans* neuronal aging transcriptomes correlate with human brain aging patterns and anticorrelate with geroprotective interventions. We performed an in silico drug screen to identify potentially neuroprotective small molecules. We show that the natural occurring plant metabolite syringic acid and the piperazine derivative vanoxerine delay neuronal degeneration, and propose these compounds as neuroprotective interventions. Furthermore, we identify neurotoxins that accelerate neurodegeneration, indicating that distinguishing aging trajectories between neuron types can inform on protective interventions as well as risk factors.

## Abstract (web summary)

Gallrein et al. pair functional assays with clocks to compare chronological versus biological age of single neuron types in *C. elegans*, to probe their vulnerability or resilience to neurodegeneration, and use an in silico screen to identify neuroprotective drugs.

## Main

Aging is the highest risk factor for neurodegenerative diseases such as Alzheimer’s disease or Parkinson’s disease that are triggered by the functional decline of distinct neuron types. In Alzheimer’s disease, the initially degenerating brain structures are the parahippocampal gyrus and the olfactory bulb, followed by the degeneration of the hippocampus, leading to the characteristic clinical dementia symptoms^1–3^. Parkinson’s disease, in contrast, affects mostly the dopaminergic innervation of the midbrain and the cerebellum, resulting in the perturbation of motility and the induction of tremor syndromes^4,5^. In mice, distinct classes of brain cells show differences in age-related decline^6^, and single-cell transcriptomics has identified pro-aging and pro-rejuvenating proximity effects of distinct cell populations^7^. The extent to which intrinsic aging susceptibility differs among individual neuron types has, however, remained largely unknown. To elucidate whether different neuron types might differ in their biological aging process, we used *C. elegans* as an experimental model with a well-characterized neuronal system of 302 neurons of ≥128 different neuron types^8^ that allows assessing the integrity of individual neurons during the normal aging process in vivo.

Aging clocks provide predictive models of molecular signatures (for example, DNA methylation) that estimate an individual’s chronological age, and could identify biological age differences resulting from genetic or pharmacological age acceleration or deceleration^9–11^. Based on the known effects of many genetic and pharmacologic interventions on lifespan and thus biological age in *C. elegans*, we recently developed the highly accurate binarized transcriptomic (Bit) age clock. BitAge predicts already in young adult animals quantitatively how genetic, environmental or pharmacological treatments affect lifespan^10^. We previously established that also the simulation of increasing noise allows the prediction of biological age regardless of organism or data type, allowing us to design the Stochastic Clock to detect age accelerating and decelerating interventions^12^.

Here, we asked whether in chronologically young animals, distinct neuron types might exhibit distinct biological ages in *C. elegans*. We show that individual neuron types in the late larval L4 stage, which directly precedes adulthood, show a distinct offset of biological age prediction despite sharing the same chronological age. We demonstrate that the age prediction corresponds to neuron-type-specific degeneration and the decline of their specific function. Neuron types with an older predicted age show early neurodegeneration, while those with a younger predicted age are preserved. The transcriptomic differences between these biologically ‘older’ and ‘younger’ neuron types indicate translation as a crucial driver of neuronal aging and pharmacologically reducing protein synthesis prevented the degeneration of the fast-aging neurons. We determined that the transcriptional patterns of the biological age differences are correlated with the transcriptional patterns of human and mouse brain aging, suggesting conserved neuronal aging mechanisms. The neuronal aging patterns anticorrelated with geroprotective interventions enabling the identification of neuronal aging modulators. Using a transcriptomic resource incorporating thousands of different compounds in human cell lines (CMAP)^13^, we identified and validated pharmacological compounds that protect the integrity of neurons in vivo. We demonstrate that the naturally occurring plant metabolite syringic acid and the piperazine derivative vanoxerine prevent neurodegeneration and propose that they could serve as neuroprotective interventions. In reverse, our approach can also identify neurotoxins that accelerate neurodegeneration. Our data suggest that the mechanisms underlying neuron-type-specific aging rates allow the identification of therapeutic interventions that could slow down neuronal aging and prevent neurodegeneration.

## Results

**Fig. 1:**
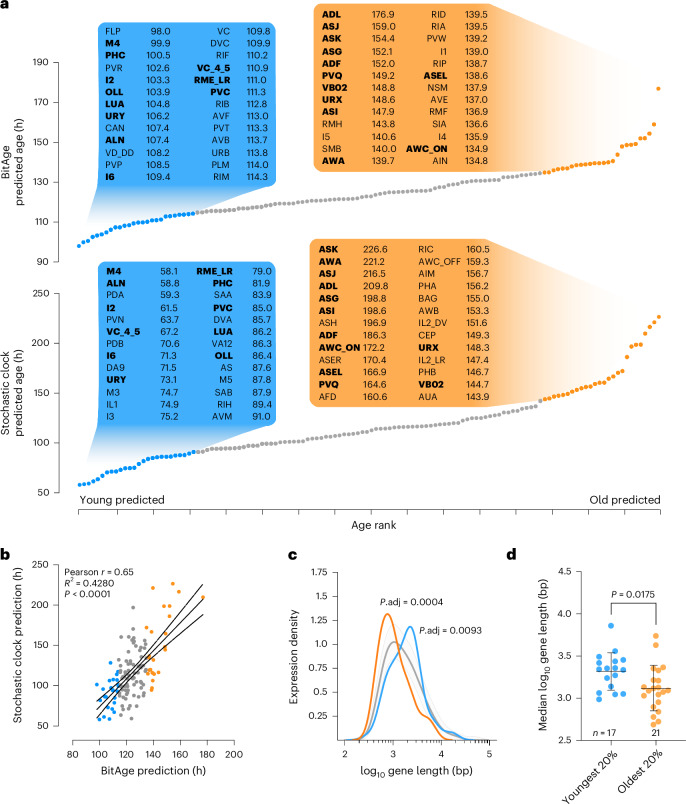
Transcriptome-based aging clocks can predict the ages of individual neuron types. **a**, Distribution of transcriptomic age predictions. The 128 neurons of the CeNGEN dataset were predicted with BitAge (upper) or a stochastic noise-based clock (lower) and sorted by their predicted age. The *x* axis shows the rank of the prediction in ascending order, and the *y* axis shows the predicted age. The 20% youngest neurons and their respective age predictions are outlined in blue; the 20% oldest neurons and their respective age predictions are displayed in orange. Neuron types appearing on the top/bottom of both prediction lists are indicated in bold letters. **b**, Predictions with BitAge and the Stochastic Clock on the CeNGEN dataset are highly correlated (Pearson correlation 0.65, *P* value 5.5 × 10^−17^). The *x* axis shows the BitAge predictions of the CeNGEN dataset, and the *y* axis shows the stochastic data-based clock predictions of the CeNGEN dataset. All 128 neurons are plotted. Color coding of the youngest 20% of neurons according to BitAge in blue and of the oldest 20% in orange. The regression model fit with a 95% confidence interval (dashed, black lines) is shown. **c**, Neuron-type-specific genes in the CeNGEN dataset are skewed depending on gene length and age prediction. The log_10_ gene length (*x* axis) of the specific genes from the 20% youngest neurons (blue), and of the 20% oldest neurons (orange) are compared to random permutation of all neuron-type-specific genes with the same number of genes (gray). The gray curve shows the mean density across 100,000 random permutations, each sampling the same number of genes as in the youngest/oldest neuron groups; the shaded gray band indicates the 95% confidence interval across permutations. The two-sided permutation test compared the median log_10_ gene length. The *y* axis shows the probability density of the values on the *x* axis. **d**, Swarm plot of the median log_10_ length of neuron-type-specific genes expressed in the youngest 20% (blue) and oldest 20% (orange) neurons according to BitAge predictions. Each point represents a single neuron type. Neuron types were grouped into the youngest 20% (blue, *n* = 17) and oldest 20% (orange, *n* = 21) according to BitAge-predicted biological age. Data shown are the mean ± s.d. Welch’s two-tailed *t*-test was used to test for significant differences. Source data

To investigate whether different neuron types age differently within an organism, we applied our previously developed BitAge biological age predictor^10^ and, as independent age predictor, our previously developed Stochastic Clock^12^ on neuron-type-specific RNA-sequencing data. We used the single-neuron-type data from the *C. elegans* Neuronal Gene Expression Map & Network (CeNGEN) dataset^14^, which comprises 128 distinct neuron types from late L4-stage larvae. The 128 neuron age predictions range from ≈98 h in FLP neurons to ≈177 h in ADL neurons (Fig. 1a; BitAge) suggesting that different neurons might show an almost twofold biological age difference in chronologically young nematodes.

**Extended Data Fig. 1:**
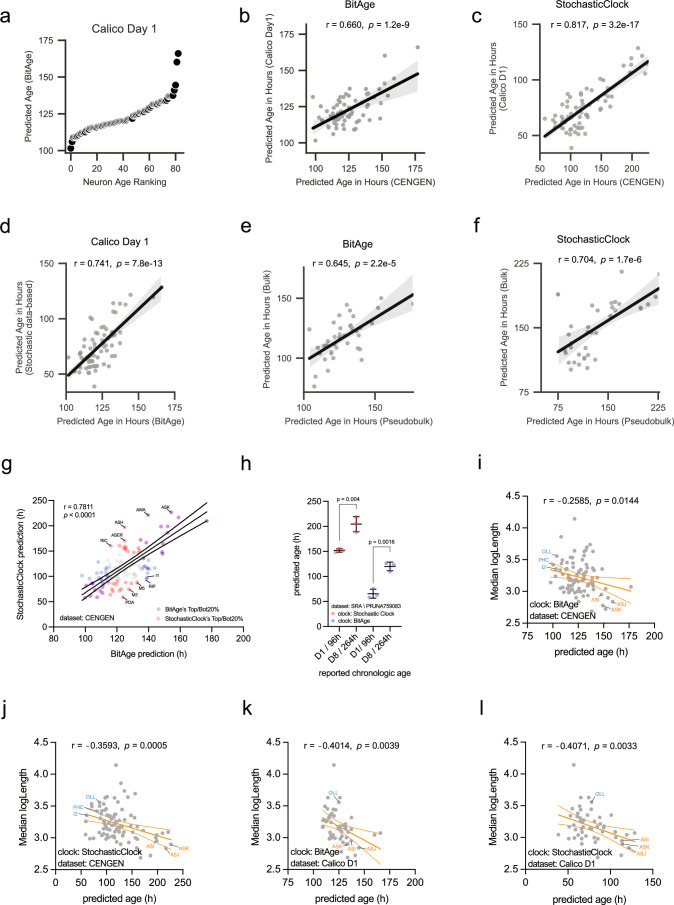
Correlations between BitAge and StochasticClock. **a**) Distribution of transcriptomic age predictions. The 67 neurons of the Calico day 1 dataset were predicted with BitAge and sorted by their predicted age. The x-axis shows the rank of the prediction is ascending order, the y-axis the predicted age. **b**) BitAge predictions on the CeNGEN and the Calico day 1 dataset are highly correlated (Pearson correlation 0.66, p-value 1.2e-09). The x-axis shows the BitAge predictions of the CeNGEN dataset, the y-axis BitAge predictions of the Calico day 1 dataset. 67 neurons are plotted. The regression model fit with a 95% confidence interval (shadowed area) is shown. **c**) Stochastic Clock predictions on the CeNGEN and the Calico day 1 dataset are highly correlated (Pearson correlation 0.82, p-value 3.2e-17). The x-axis shows the Stochastic Clock predictions of the CeNGEN dataset, the y-axis BitAge predictions of the Calico day 1 dataset. 67 neurons are plotted. The regression model fit with a 95% confidence interval (shadowed area) is shown. **d**) Age prediction of BitAge and the Stochastic Clock on Calico Day 1 data. The x-axis shows the predictions by BitAge, the y-axis the prediction by the stochastic clock. There is a strong correlation (Pearson correlation 0.74, p-value 7.8e-13). **e**) Age prediction of BitAge on CeNGEN bulk data vs CeNGEN pseudobulk. There is a strong correlation (Pearson correlation 0.65, p-value 2.2e-5). **f**) Age prediction of the Stochastic Clock on CeNGEN bulk data vs CeNGEN pseudobulk. There is a strong correlation (Pearson correlation 0.7, p-value 1.7e-6). **g**) Predictions with BitAge and the Stochastic Clock on the CeNGEN dataset are highly correlated. The x-axis shows the BitAge predictions of the CeNGEN dataset, the y-axis stochastic data-based clock predictions of the CeNGEN dataset. All 128 neurons are plotted, the 20% youngest and oldest according to BitAge are coloured in blue, the 20% youngest and oldest according to the StochasticClock are coloured in red, overlap between both violet. The remaining neurons are indicated in grey. The regression model fit with a 95% confidence interval (dashed, black lines) is shown. The five neurons the farthest away from the regression line (both, above and below) are indicated. **h**) BitAge (blue) and StochasticClock (red) predictions on an independent dataset of neuronal RNA from adult day 1 and adult day 8 old nematodes. **i**) Correlation of median gene length and predicted age of individual neuron types. BitAge clock used on the CeNGEN dataset. Linear fit (orange line) with 95% confidence interval (dashed, orange lines) is shown. Exemplary, young predicted neurons I2, OLL, and PHC (blue); and old predicted neurons ASI, ASJ, and ASK (orange) are indicated. **j**) Correlation of median gene length and predicted age of individual neuron types. Stochastic clock used on the CeNGEN dataset. Linear fit (orange line) with 95% confidence interval (dashed, orange lines) is shown. **k**) Correlation of median gene length and predicted age of individual neuron types. BitAge clock used on the day 1 Calico dataset. Linear fit (orange line) with 95% confidence interval (dashed, orange lines) is shown. **l**) Correlation of median gene length and predicted age of individual neuron types. Stochastic clock used on the day 1 Calico dataset. Linear fit (orange line) with 95% confidence interval (dashed, orange lines) is shown. Source data

We independently confirmed the different age predictions of the neuron types in the single-neuron-type RNA-sequencing data from young adults in a recent cell atlas of *C. elegans* aging (Calico)^15^, which contains 67 of the 128 neuron types from the CeNGEN dataset. Also, the Calico dataset of day 1 adults exhibits the same predicted age distribution (Extended Data Fig. 1a), and the BitAge predictions on both datasets are significantly correlated with each other (Extended Data Fig. 1b; Pearson correlation 0.66, *P* < 0.0001).

To independently assess each neuron’s biological age, we applied a Stochastic Clock that does not rely on specific transcriptomic signatures but instead simulates how random changes accumulate over time^12^. Despite using a fundamentally different strategy, the Stochastic Clock showed substantial overlap with BitAge (Fig. 1a) and predicts an almost fourfold age difference between the youngest and oldest neurons. The predictions of the Stochastic Clock significantly correlate with the BitAge predictions on the CeNGEN dataset (Fig. 1b; Pearson correlation *r* = 0.65, *P* < 0.0001), which was even stronger within the very young or very old predicted group of neurons (Extended Data Fig. 1g; Pearson correlation *r* = 0.78, *P* < 0.0001; for the top and bottom 20%). The Stochastic Clock predictions showed a significant correlation between the CeNGEN and Calico datasets (Extended Data Fig. 1c; Pearson correlation 0.82, *P* < 0.0001). Similarly, the Stochastic Clock predictions correlated significantly with BitAge in the Calico dataset (Extended Data Fig. 1d; Pearson correlation 0.74, *P* < 0.0001). Taken together, two independent aging clock paradigms using two independent single-neuron transcriptome datasets consistently predict specific neuron types as younger and others as older in chronologically young animals.

To address potential limitations of single-cell sequencing coverage, we performed the same analyses on bulk RNA-sequencing data from CeNGEN, which offers improved transcriptomic coverage for a subset of 37 neurons^16^. BitAge predictions remained consistent between the pseudobulk CeNGEN dataset and the bulk RNA-sequencing dataset (Extended Data Fig. 1e; Pearson correlation 0.65, *P* < 0.0001). Similarly, the Stochastic Clock predictions on pseudobulk and bulk CeNGEN datasets were also significantly correlated (Extended Data Fig. 1f; Pearson correlation 0.7, *P* < 0.0001), reinforcing that the predicted biological age differences among neurons are robust across different datasets and sequencing approaches. While the predictions of BitAge and the Stochastic Clock are highly consistent, there are also some outliers that deviate more strongly from the correlation (Extended Data Fig. 1g).

As additional validation, we used bulk neuron transcriptomes from young and aged animals that were not included in the training data of the clocks^17^. We found that both clocks could differentiate between the young and old samples (Extended Data Fig. 1h); note that differences between predicted biological age and indicated chronological age could arise from two effects: first, the sample age is reported in days, giving a ±12-h uncertainty; second, the associated reported median lifespan of 22 days is unexpectedly high (median lifespan is typically between 14 and 18 days)^17^.

The aging transcriptome in species ranging from *C. elegans* to humans was recently shown to exhibit a gene length-dependent transcriptional decline (GLTD): the longer the gene, the more sharply its expression tends to decrease with age^18,19^. GLTD is thought to result from the age-dependent accumulation of transcription-blocking DNA damage that is more likely to occur in larger genes. To test whether neurons predicted to be biologically older showed GLTD, we analyzed neuron-type-specific genes, which are defined as genes that are expressed in at least 90% of the specific neurons and in not more than 10% of other cells. In line with GLTD, the neuron-type-specific genes of the 20% oldest predicted neurons are significantly shorter than expected by chance (*P* = 3.6 × 10^−4^), while the neuron-type-specific genes of the 20% youngest neurons are significantly longer (*P* = 9.28 × 10^−3^; Fig. 1c) and show a significantly longer median gene length (Fig. 1d). The median length of the neuron-type-specific genes is negatively correlated with the prediction age of BitAge or the Stochastic Clock on either CeNGEN or Calico data (Extended Data Fig. 1i–l). These results indicate that the clock-predicted biological age is reflected in the age-associated GLTD that is most prevalent in the biologically old predicted neurons.

Taken together, the alignment of aging clock predictions with the presence of GLTD in neurons predicted to be biologically old in chronologically young animals provides compelling evidence that specific neuron types undergo accelerated aging.

### Neuron-specific age predictions are associated with neurodegeneration

**Fig. 2:**
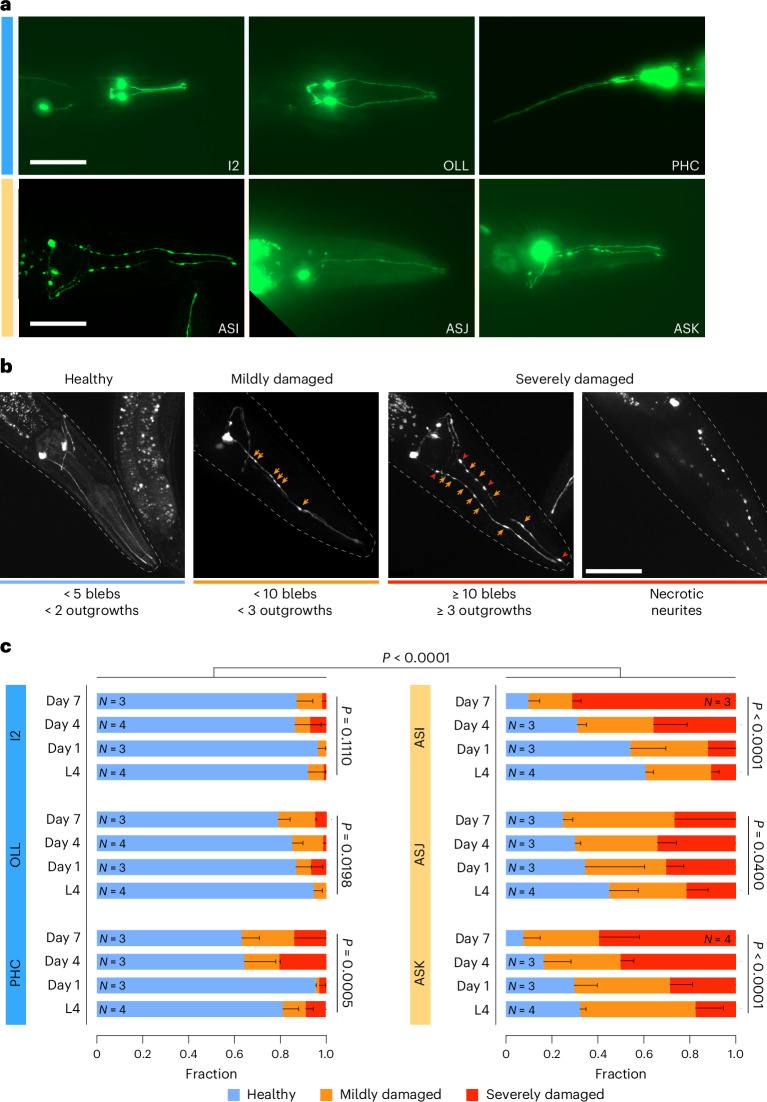
Predicted neuron age and degeneration onset and progression correlate. **a**, Representative fluorescence images (*z*-stack maximum projections) of the analyzed neurons grouped by prediction age—I2, OLL and PHC as representatives of the young neurons (blue); ASI, ASJ and ASK as representatives of the old neurons (orange). Scale bars, 50 µm. **b**, Representative fluorescence images (*z*-stack maximum projections) of nematodes expressing neuronal GFP markers, classified according to the severity of observed degeneration. Classification criteria are indicated below the images. Orange arrows indicate blebs; red arrowheads indicate spheric outgrowths. Nematode heads are outlined by a dashed line. Scale bar, 50 µm. **c**, Fraction plots displaying the fraction of nematodes expressing neuronal markers in different neurons categorized as ‘healthy’, ‘mildly damaged’ and ‘severely damaged’. Three to four cohorts were analyzed, comprising 10–30 individual nematodes, for every time point indicated. Data shown are the mean ± s.d. Statistical analysis was performed using a cumulative link model (CLM) with a logit link to account for the ordered categorical nature of neuronal damage scores (healthy < mild < severe). *P* values were adjusted for multiple testing using the Benjamini–Hochberg method. Source data

**Extended Data Fig. 2:**
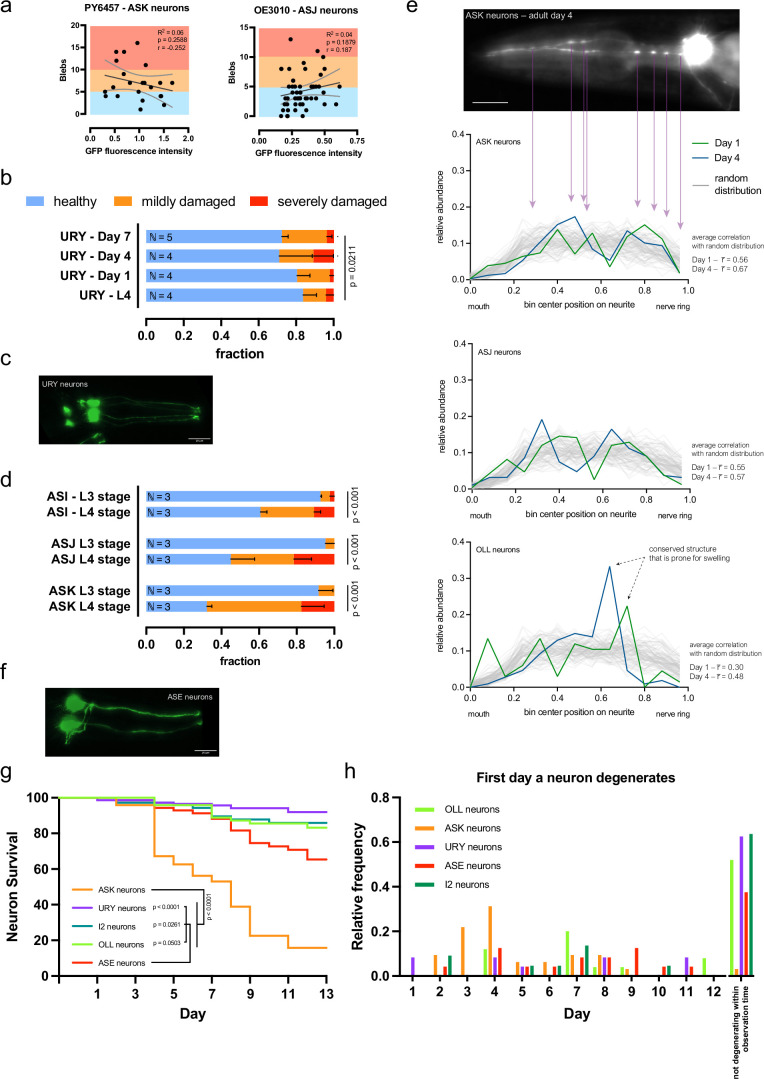
Neurite damage appearance and neuron survival analysis. **a**) Correlation of neuronal GFP expression in ASK neurons (left) or ASJ neurons (right) and scored blebs (major hallmark of neurodegeneration in these strains). Classification to the fraction of ‘healthy’ (blue), ‘mildly damaged’ (orange), or ‘severely damaged’ (red) neurons is indicated. Linear regression (black line) and confidence band (grey lines) are displayed, and no significant correlation was found. Displayed are the pooled data of four independent cohorts of 12 – 25 adult day 1 old nematodes each. **b**) Fraction plot of neuronal integrity of URY neurons upon aging. Four to five cohorts of 12 – 25 JKM10 nematodes expressing GFP in URY neurons have been recorded and neurodegeneration was assessed and neurons categorized as ‘healthy’ (blue), ‘mildly damaged’ (orange), and ‘severely damaged’ (red). Kruskal-Wallis test was performed, and no significant differences was observed. **c**) Z-Stack Maximum projection after background subtraction (Fiji – rolling ball radius 25) of fluorescence microscopic recording of representative JKM10 nematode expressing GFP in URY neurons. Scale bar indicates 20 µm. **d**) Fraction plot of neuronal integrity of ASI, ASJ, and ASK neurons at young ages (L3 and L4 stage). Three to four cohorts of 8 – 27 JKM10 nematodes expressing GFP in either ASI, ASJ, or ASK neurons have been recorded, neurodegeneration was assessed, and neurons categorized as ‘healthy’ (blue), ‘mildly damaged’ (orange), and ‘severely damaged’ (red). Ordinal regression (cumulative link model) has been used to test for significant differences between ages. **e**) Stochastic appearance of neuronal damage. The top panel shows a representative epi fluorescent image of ASK neurons. Scale bar is 20 µm. Blebs that were counted as damaged sites are indicated by violet arrows, which also depict how damage position is mirrored in the histograms below. The three bottom panels show histograms of the neuronal damage distribution at day 1 (green line) and day 4 (blue line) of the indicated neurons (ASK, ASJ, and OLL). Three cohorts of 10 – 25 individuals have been analysed per strain and age. Grey lines are 100 examples of randomly distributed damage along neurites. Damage site was measured as distance from the mouth and binned into 12 damage-site-bins. Pearson correlation has been calculated for every pair of “neuron *vs*. random distribution” and the average is indicated. **f**) Z-Stack Maximum projection after background subtraction (Fiji – rolling ball radius 25) of fluorescence microscopic recording of representative NY2067 nematode expressing GFP in ASE neurons. Scale bar indicates 20 µm. **g**) Neuron survival was assessed using Kaplan-Mayer. Presence of green fluorescent neuron bodies was scored daily. Mantel-Cox test was done to test for significant differences. Shown are the aggregated plots of three independent cohorts of each 12 nematodes per strain. **h**) Histogram showing the relative frequency of the first day of neuron loss. Three cohorts of each 12 nematodes per strain. Source data

To assess whether the predicted age differences are associated with different degrees of neuron-specific degeneration, we next chose three young (I2, OLL, PHC) and three old (ASI, ASJ, ASK) predicted neurons (Fig. 2a) and scored their degeneration over the chronological age starting from the larval L4 stage onward to day 7 of adulthood. The L4 stage is the last larval stage before the nematodes reach sexual maturity, while day 7 of adulthood is in the post-fertile phase. To assess the morphological integrity of specific neurons in vivo, we used neuron-type-specific transcriptional green fluorescent protein (GFP) reporters (Fig. 2a). Macroscopic aberrations in the neurites were classified as ‘healthy’, ‘damaged’ or ‘severely damaged’ (Fig. 2b) as established previously^20–23^. We excluded that GFP expression levels might influence neuron integrity, as GFP expression levels and neuronal integrity showed no correlation as tested in ASK and ASJ neurons that exhibit the strongest variance in GFP expression levels among the employed nematode strains (Extended Data Fig. 2a).

In accordance with our predictions, the three young predicted neuron types exhibited significantly less degeneration than the old predicted neuron types at all analyzed time points (Fig. 2c). At L4 stage and on the first day of adulthood, I2, OLL and PHC neurons exhibited a minimal degeneration only affecting 10–20% of the animals, which increased to ≈35% during aging. URY neurons, which were described previously to exhibit approximately 35% degeneration at day 7 of adulthood^23^, displayed ≈20% degeneration in L4 and young adults, which increased up to ≈35% on day 7 of adulthood (Extended Data Fig. 2b,c). All analyzed neuron types except I2 showed a significant age-dependent deterioration (Fig. 2c).

The predicted biologically older ASI, ASJ and ASK neurons were damaged in >45% of L4 larvae reaching up to 90% at day 7 of adulthood, indicative of rapid aging. To determine at which stage the rapidly aging neurons start to degenerate, we also examined the ASI, ASJ and ASK neurons in the larval L3 stage. Consistent with a fully developed neuronal system, those neuron types were intact in more than 90% of the L3 animals, suggesting that the first burst of neuronal aging occurs already between the L3 and L4 stage (Extended Data Fig. 2d).

To discern whether the progressive degenerative process reflects an age-dependent stochastic process or instead a deterministic developmental program, we analyzed whether the bleb patterns appeared randomly along the neurites. The positions of blebs in ASJ, ASK and OLL neurons in adults indeed correlated with a random distribution of damage sites along neurites. Only one site in OLL neurons exhibited nonrandom bleb clustering; it was located at the proximal pharyngeal bulb and prone to swelling (Extended Data Fig. 2e).

To address whether we might have underestimated the neurodegeneration because only the morphology of neurons that express GFP could be analyzed, and hence are present, we assessed neuronal survival in individual nematodes over a time course of 12 days during adulthood. During aging most animals retained the young predicted I2, OLL and URY neurons, while the old predicted ASK and ASE neurons were progressively lost (Extended Data Fig. 2f–h), showing that early biological age predictions are associated with age-dependent neuronal attrition.

Taken together, these results validate our neuron-type-specific biological age predictions for identifying neurons at higher risk of degeneration, with potential implications for understanding selective neuronal vulnerability in aging and neurodegeneration.

### Neuronal degeneration corresponds to neuron-specific functional loss

To test whether the morphological neurite degeneration corresponds with functional impairment, we monitored behaviors that depend specifically on a single neuron type. The requirement of a highly neuron-type-specific phenotypic outcome excluded the functional analysis of the ASI, ASJ, ASK, I2, OLL and PHC neurons for any of the following reasons: (1) some neurons, such as I2, lack well-defined functional roles; (2) some functions are executed through multiple redundantly operating neurons thus precluding assessment of single-neuron function; (3) some neurons are negative regulators of other neurons leading to indirect effects. In contrast, URY neurons (representing young predicted neurons) are strictly required for selected pathogen avoidance^24^ and ASE neurons (representing old predicted neurons) mediate salt sensation and memory formation^25^ allowing their functional analysis by behavioral testing.

**Fig. 3:**
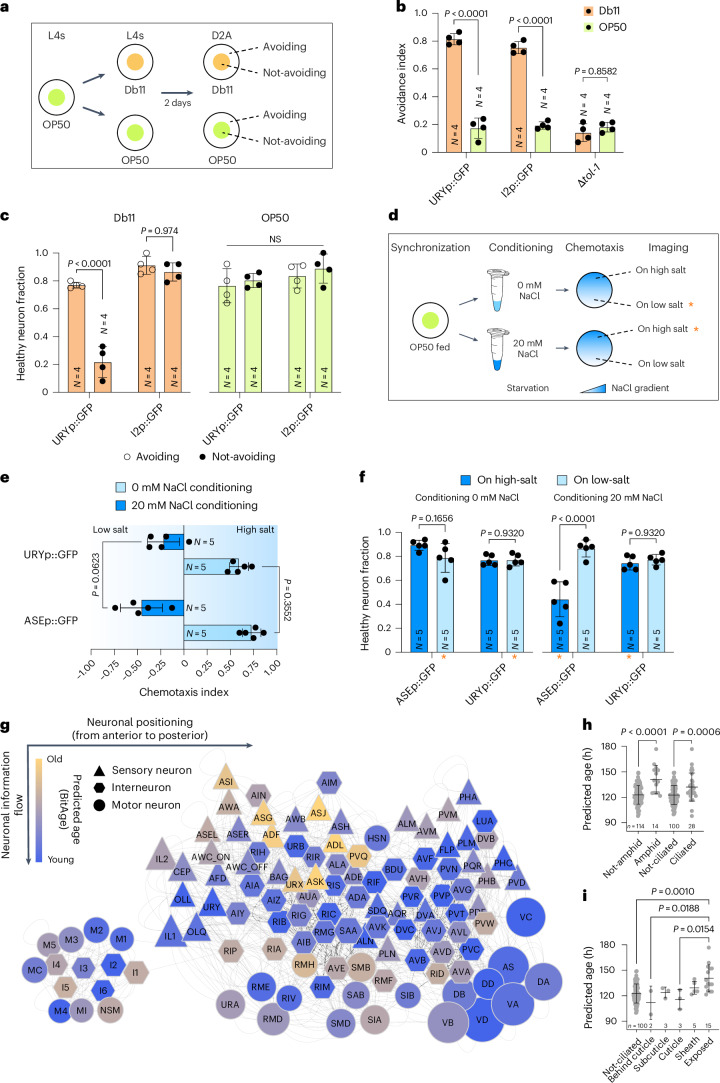
Neurodegeneration and neuronal function loss are correlated. **a**, Schematic depiction of the experimental design to examine the correlation between pathogen avoidance from *S. marcescens* Db11 bacteria and URY neuron integrity. Nematodes were synchronized by L4 picking and transferred to Db11 or OP50 bacteria. After 2 days, avoidance behavior was scored and nematodes classified as avoiding/not-avoiding and subjected to microscopy to analyze URY neuron neurite health. **b**, Swarm plot of the results from the pathogen avoidance assay from Db11 bacteria. URY neuron marker strain (URYp::GFP, JKM10), I2 neuron marker strain (I2p::GFP, MT21910, control neuron unrelated to avoidance reaction) and TOL-1 receptor mutant strain (∆*tol-1*, IG10, no avoidance from Db11) were used. Data shown are the mean ± s.d. from four independent cohorts of 50–75 nematodes. Two-way analysis of variance (ANOVA) + Tukey post hoc test were used. **c**, Swarm plot displaying the healthy neuron fraction size from nematodes subjected to Db11 pathogen or OP50 control bacteria and classified for evasion behavior. URY neuron marker strain (URYp::GFP, JKM10, mediating avoidance from *S. marcescens*) and I2 neuron marker strain (I2p::GFP, MT21910, control neuron unrelated to avoidance reaction) were used. Data are the mean ± s.d. of four independent cohorts of 10–20 nematodes per strain, condition and classification. Statistical analysis was performed using a CLM. *P* values were adjusted for multiple testing using the Benjamini–Hochberg method. **d**, Schematic depiction of the experimental design to correlate salt aversive conditioning and neurodegeneration of ASE neurons. Adult 1-day-old nematodes were conditioned by starvation in the presence or absence of NaCl, and thereafter subjected to a chemotaxis assay on an NaCl gradient. Subsequently, nematodes were classified (dwelling on high/low salt) and neurite integrity was analyzed using fluorescence microscopy. Orange asterisks indicate nematodes that failed at learning. **e**, Swarm plot showing the chemotaxis index of nematodes conditioned at 0 mM (light blue) or 20 mM (dark blue) NaCl. ASE neuron marker strain (ASEp::GFP, NY2067, mediating salt aversive conditioning) and URY neuron marker strain (URYp::GFP, JKM10, control neuron unrelated to salt aversive conditioning) were used. Data shown are the mean ± s.d. from five independent cohorts of 80–150 nematodes. Statistical analysis was performed using a CLM. *P* values were adjusted for multiple testing using the Benjamini–Hochberg method. **f**, Swarm plot displaying the healthy neuron fraction size from nematodes after salt aversive conditioning. Nematodes were grouped depending on the conditioning and their subsequent preference for a high (dark blue) or low (light blue) salt concentration. The groups that failed to connect the starvation stimulus and salt concentration are indicated by orange asterisks. ASE neuron marker strain (ASEp::GFP, NY2067, mediating salt aversive conditioning) and URY neuron marker strain (URYp::GFP, JKM10, control neuron unrelated to salt aversive conditioning) were used. Data shown are the mean ± s.d. from five independent cohorts of 10–20 nematodes. Two-way ANOVA + Tukey post hoc test were used. **g**, We adapted a previously published connectome of *C. elegans*^37^. Only neuronal cells are shown in a largely directional information flow on the vertical axis, with sensory neurons (triangles) on top, interneurons (hexagons) in the middle and motor neurons (circles) on the bottom. The horizontal axis roughly shows the anatomical orientation with the head region on the left, and posterior neurons on the right. Chemical synapses and gap junctions are indicated as faded gray lines. The size of the neurons indicates the number of cells within this neuron type. The predicted age (BitAge based on the CeNGEN dataset) is color coded from blue (young) to orange (old). The oldest neurons cluster in the middle top part and are largely sensory neurons. **h**, Swarm plot showing BitAge predictions grouped by amphid neurons/non-amphid neurons and ciliated neurons/non-ciliated neurons. Each point represents a single neuron type (*n* = 14 amphid, *n* = 114 not-amphid; *n* = 28 ciliated, *n* = 100 not-ciliated). Data shown are the mean ± s.d. A two-sided *t*-test was used. **i**, Swarm plot showing ciliated neurons’ age predictions divided into five classes depending on where their cilia terminate. Each point represents a single neuron type (*n* = 100 not-ciliated, *n* = 15 exposed, *n* = 5 sheath, *n* = 3 cuticle, *n* = 3 subcuticle, *n* = 2 behind cuticle). Data shown are the mean ± s.d. ANOVA + Tukey post hoc test were used. Source data

We tested whether the degeneration of URY neurons correlates with impaired avoidance behavior from pathogenic *Serratia marcescens* Db11 bacteria, which is primarily mediated by the TOL-1 receptor expressed in URY neurons^24^. URY-marked strains (GFP is expressed specifically in URY neurons) were exposed to either pathogenic Db11 or nonpathogenic OP50 control bacteria. To test whether failure to display avoidance behavior correlated with neurodegeneration, we assessed the integrity of the URY neurons in animals that either avoided or failed to avoid the bacterial lawn (Fig. 3a).

Approximately 80% of the URY neuron-marked animals avoided the toxic Db11 bacterial lawn thus showing a typical pathogen avoidance behavior (Fig. 3b). To ascertain the specificity of the assay, we tested a *tol-1* mutant control strain that failed to show any avoidance. A baseline avoidance of ≈20% was observed on nonpathogenic OP50, consistent with natural exploratory behavior. Nematodes that failed to avoid pathogenic Db11 showed significantly increased degeneration of URY neurons compared to those that successfully avoided the bacteria (Fig. 3c). As a control, we ascertained that degeneration of I2 neurons, which are also predicted to be biologically young (Figs. 1a and 2c) but are not involved in pathogen avoidance, was uncorrelated with the avoidance behavior, confirming that the loss of avoidance behavior was dependent on URY neuron degeneration (Fig. 3c).

To corroborate our findings, we tested salt aversive memory formation that is specifically mediated by ASE neurons^25^, which are predicted to be biologically old (Fig. 1a). Nematodes were conditioned to associate either low (0 mM) or high (20 mM) sodium chloride concentrations with starvation. Thereafter, the behavioral responses were tested by using a chemotaxis preference assay on a sodium chloride gradient and subjected to neurite imaging of either salt-sensing ASE neurons or salt-sensing unrelated URY neurons (Fig. 3d). The strains expressing GFP in either ASE or URY neurons similarly learned to move toward higher salt concentrations when a low salt concentration was associated with starvation (Fig. 3e), while nematodes conditioned to associate a high salt concentration with starvation moved toward lower salt concentrations on the salt gradient (Fig. 3e). Nevertheless, some individual nematodes failed to show the expected avoidance behavior and moved toward the salt concentration they should have learned to avoid (Fig. 3d,f). The neuronal health was assessed as described above (Fig. 2b), and the healthy neuron fraction was quantified across four behavioral groups: Nematodes (1) trained to avoid low salt (left) and found on low salt (light blue); (2) trained to avoid low salt (left) and found on high salt (dark blue); (3) trained to avoid high salt (right) and found on low salt (light blue); and (4) trained to avoid high salt (right) and found on high salt (dark blue). There was no significant difference in the healthy neuron fraction among animals conditioned on 0 mM NaCl, regardless of their behavioral outcome (Fig. 3f). In contrast, the ASE neuron-marked animals conditioned to avoid 20 mM NaCl, exhibited a significant increase in damaged neurons in the group that was moving toward the high salt concentration. As expected, URY neuron-marked nematodes did not show an accumulation of neurodegeneration in any of the groups (Fig. 3f).

In conclusion, these results establish a direct link between neuronal degeneration and functional impairment, demonstrating the power of transcriptome clock-predicting biologically older neurons (henceforth, referred to as ‘biologically old’ or ‘biologically aged’).

### Ciliated sensory neurons exposed to the environment are most rapidly aging

**Extended Data Fig. 3:**
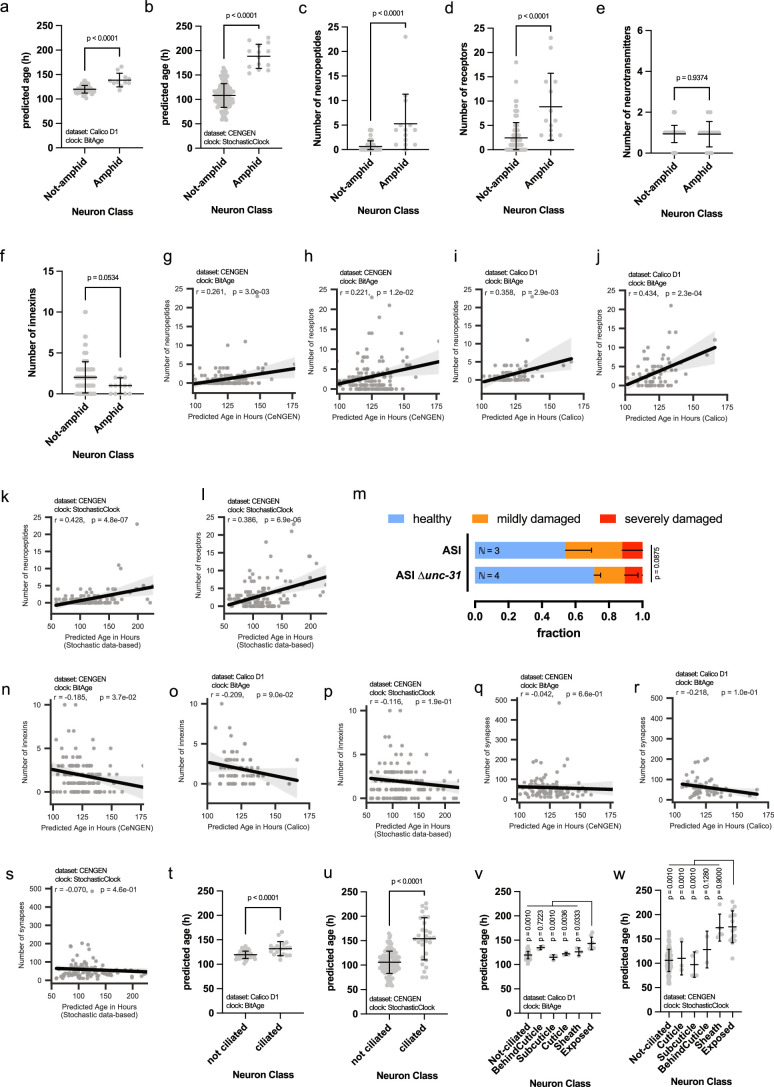
Correlations between CENGEN and Calico datasets. **a**) Amphid neurons are predicted to be significantly older than non-amphid neurons in the Calico day 1 dataset. Two-sided t-test p-value: 5.6e-08. **b**) Amphid neurons are predicted to be significantly older than non-amphid neurons in the CeNGEN dataset with a stochastic data-based clock. Two-sided t-test p-value: 7.2e-22. **c**) Amphid neurons express significantly more neuropeptides than non-amphid neurons. Two-sided t-test p-value: 5.95e-12. **d**) Amphid neurons express significantly more receptor genes than non-amphid neurons. Two-sided t-test p-value:8.45e-09. **e**) The number of neurotransmitters is not significantly different in amphid and non-amphid neurons. Two-sided t-test p-value: 0.94. **f**) The number of innexins is not significantly different in amphid and non-amphid neurons. Two-sided t-test p-value: 0.05. **g**) The number of expressed neuropeptides (y-axis) is significantly correlated (Pearson correlation 0.26, p-value 3e-03) with the predicted age by BitAge in the CeNGEN dataset. The regression model fit with a 95% confidence interval (shadowed area) is shown. **h**) The number of expressed receptor genes (y-axis) is significantly correlated (Pearson correlation 0.22, p-value 1.2e-02) with the predicted age by BitAge in the CeNGEN dataset. The regression model fit with a 95% confidence interval (shadowed area) is shown. **i**) The number of expressed neuropeptides (y-axis) is significantly correlated (Pearson correlation 0.36, p-value 2.9e-03) with the predicted age by BitAge in the Calico day 1 dataset. The regression model fit with a 95% confidence interval (shadowed area) is shown. **j**) The number of expressed receptor genes (y-axis) is significantly correlated (Pearson correlation 0.44, p-value 2.4e-04) with the predicted age by BitAge in the Calico day 1 dataset. The regression model fit with a 95% confidence interval (shadowed area) is shown. **k**) The number of expressed neuropeptides (y-axis) is significantly correlated (Pearson correlation 0.43, p-value 4.8e-07) with the predicted age by a stochastic data-based clock in the CeNGEN dataset. The regression model fit with a 95% confidence interval (shadowed area) is shown. **l**) The number of expressed receptor genes (y-axis) is significantly correlated (Pearson correlation 0.39, p-value 6.9e-06) with the predicted age by a stochastic data-based clock in the CeNGEN dataset. The regression model fit with a 95% confidence interval (shadowed area) is shown. **m**) Fraction-plot displaying the fraction of nematodes expressing neuronal volume markers in the ASI neuron categorized as ‘healthy’, ‘mildly damaged’, and ‘severely damaged’. Three to four cohorts were analysed, comprised of 10 – 30 individual nematodes, for every timepoint indicated. Kruskal-Wallis-test was employed to test for significant differences. **n**) The number of expressed innexin genes (y-axis) is significantly anti-correlated (Pearson correlation -0.19, p-value 3.7e-02) with the predicted age by BitAge in the CeNGEN dataset. The regression model fit with a 95% confidence interval (shadowed area) is shown. **o**) The number of expressed innexin genes (y-axis) is not-significantly anti-correlated (Pearson correlation -0.2, p-value 0.9e-01) with the predicted age by BitAge in the Calico day 1 dataset. The regression model fit with a 95% confidence interval (shadowed area) is shown. **p**) The number of expressed innexin genes (y-axis) is not-significantly anti-correlated (Pearson correlation -0.12, p-value 1.9e-01) with the predicted age by a stochastic data-based clock in the CeNGEN dataset. The regression model fit with a 95% confidence interval (shadowed area) is shown. **q**) The number of total synapses (y-axis) is not-significantly anti-correlated (Pearson correlation -0.04, p-value 6.6e-01) with the predicted age by BitAge in the CeNGEN dataset. The regression model fit with a 95% confidence interval (shadowed area) is shown. **r**) The number of total synapses (y-axis) is not-significantly anti-correlated (Pearson correlation -0.21, p-value 1.0e-01) with the predicted age by BitAge in the Calico day 1 dataset. The regression model fit with a 95% confidence interval (shadowed area) is shown. **s**) The number of total synapses (y-axis) is not-significantly anti-correlated (Pearson correlation -0.07, p-value 4.6e-01) with the predicted age by a stochastic data-based clock in the CeNGEN dataset. The regression model fit with a 95% confidence interval (shadowed area) is shown. **t**) Ciliated neurons are predicted to be significantly older than non-ciliated neurons in the Calico day 1 dataset. Two-sided t-test p-value: 4.4e-05. **u**) Ciliated neurons are predicted to be significantly older than non-ciliated neurons in the CeNGEN dataset with a stochastic data-based clock. Two-sided t-test p-value: 2.29e-14. **v**) Ciliated neurons are divided into 5 classes depending on where its cilia terminate. Neurons with exposed cilia are significantly older than non-ciliated neurons or neurons which cilia terminate in the cuticle or behind the cuticle in the Calico day 1 dataset (one-way ANOVA p-value: 2.68e-08, with a post-hoc Tukey test). **w**) Ciliated neurons are divided into 5 classes depending on where its cilia terminate. Neurons with exposed cilia are significantly older than non-ciliated neurons or neurons which cilia terminate in the cuticle or behind the cuticle in the CeNGEN dataset with a stochastic data-based clock (one-way ANOVA p-value: 6.6e-18, with a post-hoc Tukey test). Source data

To understand commonalities among the biologically young, as well as among the rapidly aging neuron types, we adapted a hierarchical whole-animal connectome for *C. elegans*^26^ with a rough anatomical correspondence on the *x* axis and directional flow of neuronal signaling on the *y* axis and color coded it with the predicted biological age (Fig. 3g). The biologically oldest neurons clustered in the upper-middle part of the network and consist mostly of sensory neurons, while the youngest neurons clustered further to the right. Of the 10 oldest neurons, 6 are amphid neurons (ADL, ASJ, ASK, ASG, ADF, ASI), the primary chemosensory organ, which is mostly ciliated^27^. The 14 amphid neurons of the CeNGEN dataset showed a significantly increased biological age compared to the other 114 neurons (Fig. 3h), which can be replicated in the Calico dataset (Extended Data Fig. 3a), and with the Stochastic Clock (Extended Data Fig. 3b). The sensory amphid neurons express a variety of neuropeptides, neurotransmitters, receptors and innexins to transmit the sensed cues. The numbers of expressed neuropeptides and receptors were significantly higher in amphid neurons (Extended Data Fig. 3c,d), while the number of neurotransmitter or innexins was not significantly changed (Extended Data Fig. 3e,f)^28^. Moreover, the numbers of neuropeptides and receptors per neuron were significantly positively correlated with the predicted biological age in the CeNGEN dataset (Extended Data Fig. 3g,h), the Calico dataset (Extended Data Fig. 3i,j) and the Stochastic Clock predictions (Extended Data Fig. 3k,l). To test whether neuropeptide release promotes neuronal aging, we used *unc-31* mutants defective in neuropeptide release and observed a mild, yet nonsignificant, reduction in ASI degeneration (Extended Data Fig. 3m). The number of innexins and number of total synapses per neuron did not show a significant correlation with biological age (Extended Data Fig. 3n–s).

Amphid neurons are part of the ciliated neuron classes; comparing all 28 ciliated neurons with the remaining 100 neurons also shows a significantly increased biological age (Fig. 3h), which can be replicated in the Calico dataset (Extended Data Fig. 3t) and with the Stochastic Clock (Extended Data Fig. 3u). The ciliated neurons can be further divided into five distinct classes depending on where their cilia terminate^27^. Neurons with cilia exposed to the environment show the highest biological age (Fig. 3i), while the other ciliated neuron classes are not significantly different from not-ciliated neurons. A similar effect can be observed in the Calico dataset (Extended Data Fig. 3v) and with the Stochastic Clock (Extended Data Fig. 3w). These results indicate that the oldest neurons are functionally related and mostly consist of ciliated sensory neurons that have contact with the environment and produce neuropeptides.

### Transcriptional clustering identifies translation to promote neuronal aging

**Fig. 4:**
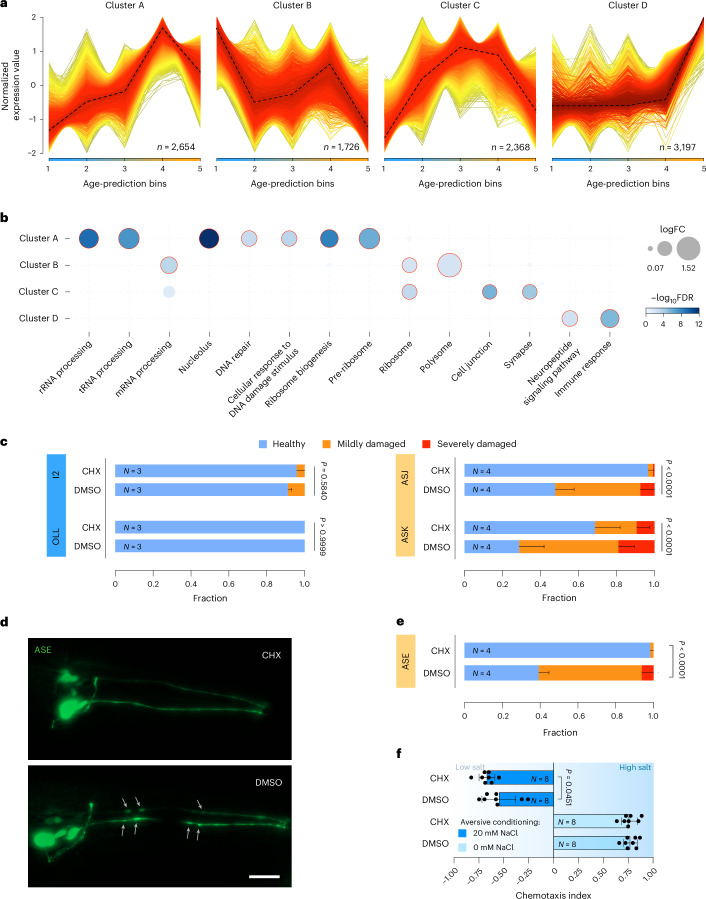
Fuzzy clustering reveals translation dynamics as a potential driver of neuron aging. **a**, Fuzzy clustering on *z*-score-normalized gene expression values (*y* axis) over the predicted aging course of the 128 CeNGEN neurons identified four clusters. The 128 neurons were merged into five age-prediction bins: (1) 97–110 h, (2) 110–120 h, (3) 120–130 h, (4) 130–140 h and (5) 140–180 h (*x* axis). The number of genes within each cluster are annotated. Dashed black lines show the central trend. **b**, Pathway overrepresentation analysis of the four clusters shows age-related pathways. The *x* axis shows pathway terms, and the *y* axis indicates clusters. Circle size reflects the enrichment score, defined as the log_2_ ratio of observed-to-expected genes per pathway (log_2_ enrichment ratio). Circle color represents statistical significance as –log_10_(false discovery rate (FDR)). Only representative nonredundant pathways are shown for visualization (Methods). **c**, Fraction plots displaying the fraction of nematodes at day 1 of adulthood expressing GFP in different neurons categorized as ‘healthy’, ‘mildly damaged’ and ‘severely damaged’ that were treated with 2 mM CHX for 24 h. Three to four cohorts were analyzed, consisting of 10–30 individual nematodes. Data shown are the mean ± s.d. Ordinal regression (CLM) was used to test for significant differences. **d**, Representative fluorescence microscopy images (*z*-stack maximum projections) of ASE neurons in 1-day-old adult nematodes of strain NY2067 after 24-h treatment with 2 mM CHX or dimethylsulfoxide (DMSO) control. Gray arrows indicate morphologic aberrations along the neurites. Scale bar, 20 µm. **e**, Fraction plots displaying the fraction of nematodes expressing GFP in ASE neurons categorized as ‘healthy’, ‘mildly damaged’ and ‘severely damaged’ that were treated with 2 mM CHX for 24 h at day 1 of adulthood. Four cohorts were analyzed, consisting of 10–30 individual nematodes. Data shown are the mean ± s.d. Ordinal regression (CLM) was used to test for significant differences. **f**, Swarm plot showing the chemotaxis index of nematodes conditioned at 0 mM (light blue) or 20 mM (dark blue) NaCl. The ASE neuronal marker strain (NY2067, mediating salt aversive conditioning) was incubated with 2 mM CHX or DMSO control for 24 h at day 1 of adulthood. Data shown are the mean ± s.d. from eight independent cohorts of 20–100 nematodes. A one-tailed *t*-test was used to test for significant differences. Source data

**Extended Data Fig. 4:**
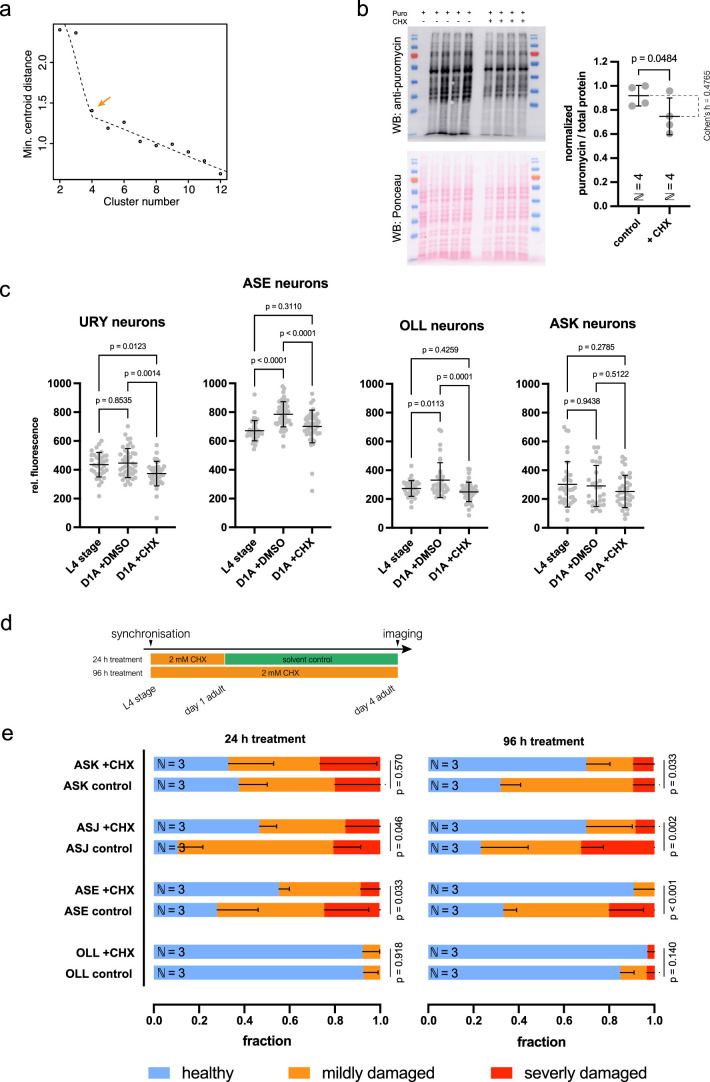
CHX is reducing translation in nematodes. **a**) Output of the Dmin function of the Mfuzz R package. Soft clustering for cluster numbers ranging from 2-12 (x-axis) were calculated with Dmin. For each cluster number, Dmin calculates the distance between the centroids of the clusters (centroid distance) and reports the minimum centroid distance across 3 repetitions (y-axis). The optimal cluster number is estimated from the “elbow” of the plot (indicated by an orange arrow on the dashed line), that is the cluster number which shows a sharp decline in the minimum centroid distance (cluster Number=4). **b**) SUnSET assay to test translation efficacy upon cycloheximide treatment. Left side: Westernblot detecting puromycin labelled proteins, ponceau staining as loading control, left side: densitometric quantification normalized to total protein according to ponceau signal. Four independent cohorts of each ≈ 35 nematodes tested. One-tailed t-test to test for significance. Lysed nematodes were day 1 of adulthood, 2 mM CHX treatment since L4 stage and while puromycin labelling. **c**) Fluorescence quantification to assess GFP synthesis in selected neurons as proxy for translation. Three independent cohorts, total 28 – 52 nematodes analysed per strain and condition. One-way ANOVA + Tukey post hoc test. **d**) Schematic depiction of experimental procedure to test the effect of either short-term or chronic CHX treatment on the neuron integrity. **e**) Fraction plots of neuronal integrity of ASK, ASJ, ASE, and OLL neurons treated with 2 mM CHX for either 24 h or 96 h starting from L4 stage on. Three cohorts of 4 – 20 nematodes expressing GFP in either ASK, ASJ, ASE, or OLL neurons have been recorded, neurodegeneration was assessed, and neurons categorized as ‘healthy’ (blue), ‘mildly damaged’ (orange), and ‘severely damaged’ (red). Ordinal regression (cumulative link model) has been used to test for significant differences between CHX and solvent control treatment. To test for significant differences a cumulative link model was built, pairwise comparison was corrected according to Bonferroni. Source data

To identify the transcriptional patterns and signatures underlying the biological age distinctions, we initially categorized the 128 neuron types into five distinct groups based on their biological age ranking (for example, the 25 youngest in group 1, position 26 to 50 in group 2). A fuzzy clustering analysis of 4 transcriptional clusters (determined by the standard elbow criterion to provide the best balance between cluster resolution and interpretability) identified neuronal age-dependent gene expression trends (Fig. 4a and Extended Data Fig. 4a).

Cluster A is composed of genes that generally increased over biological age, and is enriched for genes involved in nucleolar functions such as rRNA and tRNA processing, ribosome biogenesis and pre-ribosomes, as well as DNA repair (Fig. 4b; cluster A). Cluster B, composed of genes with highest expression in the youngest age group, an overall decline over the biological age time course including some variation and a peak in age group 4, is enriched in mRNA processing and ribosomal genes. Cluster C, comprising genes increasingly expressed over the predicted age and then sharply declining in the oldest age group, is enriched for cell junctions and synapse-related genes. Cluster D contains genes that only strongly increase in the oldest age group with functions in neuropeptide signaling and immune response. In sum, these four clusters suggest roles for translation, ribosome biogenesis and synaptic function in neuronal aging.

### Inhibition of translation alleviates neurodegeneration in fast-aging neurons

Based on the enrichment of protein biosynthesis processes in the accelerated aging neurons, we tested whether translational activity contributes to neurodegeneration. To reduce protein biosynthesis, we treated nematodes with the translation inhibitor cycloheximide (CHX) at a dose that induces translation reduction but not full inhibition^29^. Here, we scored neurite degeneration in biologically older ASK and ASJ neurons, as well as in biologically younger I2 and OLL neurons. To confirm reduction of translation, we used a SUnSET assay and observed that CHX treatment significantly reduced overall protein synthesis (Extended Data Fig. 4b) with a Cohen’s *h* of 0.48, indicating a mild reduction of translation. Additionally, we observed reduced translation in individual neurons as assessed by the reduction of GFP expression in the presence of CHX in three of the four tested neuron types. These results further corroborate our approach using CHX treatment to reduce translation also in the analyzed neurons (Extended Data Fig. 4c).

In the biologically young neurons, no effect of CHX treatment was observed (Fig. 4c). In contrast, biologically old neurons exhibited significantly less neurite deterioration upon CHX treatment (Fig. 4c). Consistently, ASE neurons that were CHX treated showed significantly less blebs than solvent control-treated neurons (Fig. 4d,e), and the nematodes showed an improved salt evasion response after they were conditioned to associate high salt concentrations with starvation (Fig. 4f), indicative of preserved neuronal health. Furthermore, we tested whether CHX treatment would have a lasting effect on neuronal integrity (Extended Data Fig. 4d,e). Even at adult day 4, ASJ and ASE neurons showed significantly improved health when treated with CHX for only 24 h beginning at the L4 stage. Chronic treatment for the whole duration until neurite damage scoring conferred even stronger neuroprotection. Taken together, these results indicate that translational activity promotes neurodegeneration and that reduced translation could restore neuronal integrity.

### Neuronal aging patterns in *C. elegans* show similarity to human brain aging

**Fig. 5:**
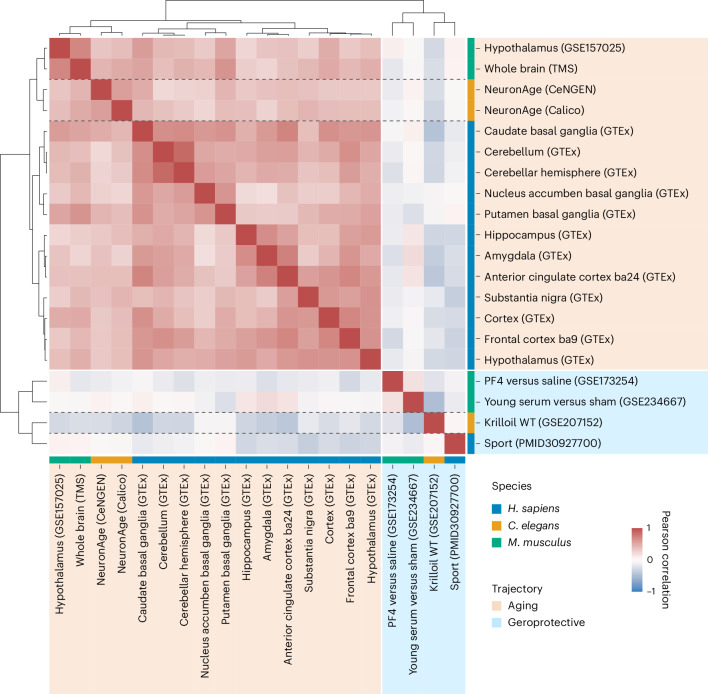
Neuronal aging patterns are conserved across *C. elegans*, mice and humans. The normalized enrichment scores of the conserved KEGG pathways for the indicated aging pattern or treatment effects were used for an unbiased clustering analysis. The matrix is color coded according to the Pearson correlation between the indicated comparisons. The colors on the side indicate the species and whether it was an aging trajectory or a geroprotective treatment. Source data

**Extended Data Fig. 5:**
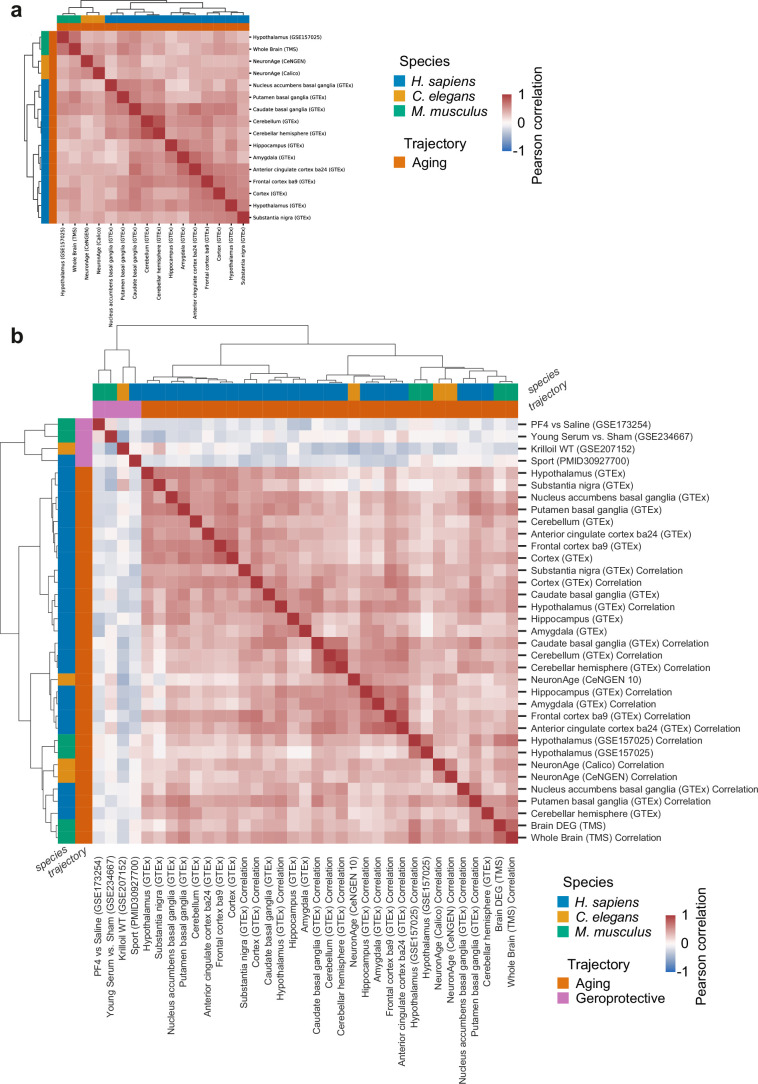
Clustering of enrichment scores for pathway and gene expression level clustering analysis. **a**) The normalized enrichment scores of the conserved KEGG pathways for the indicated aging pattern were used for an unbiased clustering analysis (the same as Fig. 5, but focusing on the aging pattern). The matrix is color-coded according to the Pearson correlation between the indicated comparisons. The colors on the side indicate the species. **b**) Unbiased clustering analysis of conserved KEGG pathway enrichments for aging patterns (*C. elegans* NeuronAge, mouse, human) and geroprotective interventions. Both Pearson correlation-based and log-fold change (logFC)-based pathway enrichments are shown for each aging dataset to assess the impact of preprocessing methodology. Despite differences in computational approach, aging datasets cluster together, while geroprotective interventions form a separate cluster, reinforcing the robustness of the observed transcriptomic relationships. The colors on the side indicate the species and whether it was an aging trajectory or a geroprotective treatment. Source data

Next, we addressed whether the biological age-related transcriptional patterns of neurons in young adult *C. elegans* (NeuronAge) are conserved in higher organisms. We defined NeuronAge as the transcriptome changes across the predicted age, quantified by the Pearson correlation between the *z*-scored transcripts per million (TPM) values and the predicted ages of 128 neurons. We then compared the conserved Kyoto Encyclopedia of Genes and Genomes (KEGG) pathway enrichments of NeuronAge with mouse and human brain aging datasets. We computed age correlations of *z*-score-normalized gene counts for all human brain regions during aging in the GTEx dataset, the Tabula Muris Senis (TMS) dataset and an additional mouse hypothalamus aging cohort (GSE157025). Similarly, we calculated the enriched pathways in brain datasets for several geroprotective treatments such as young serum injections, the platelet factor PF4, sport in humans and krill oil in *C. elegans* (for references, see the Methods). An unbiased clustering analysis revealed that the transcriptomic NeuronAge pattern of *C. elegans* and the chronological brain aging trajectories of mouse and humans cluster together (Fig. 5 and Extended Data Fig. 5a). We validated the clustering of NeuronAge by including the conserved pathway enrichments for NeuronAge in the Calico dataset. Both NeuronAge and its Calico validation showed significant positive correlations with all individual brain aging trajectories of humans and mice after multiple testing correction. The transcriptomic pattern of the geroprotective interventions formed a separate cluster that negatively correlates with the brain aging pattern, irrespective of the organism. To address potential methodological differences between aging and geroprotective datasets, that is, that aging patterns were derived using Pearson correlations with age, whereas geroprotective effects were analyzed using log-fold changes (logFC), we additionally computed logFC-based pathway enrichments for all aging datasets by comparing the oldest and youngest sample groups within each dataset. The resulting clustering (Extended Data Fig. 5b) confirmed that aging-related patterns remain distinct from geroprotective interventions, regardless of whether Pearson correlation or logFC-based methods were applied. These results indicate that neuronal transcriptomic aging patterns are conserved from nematodes to humans and that known geroprotective treatments anticorrelate with the aging datasets supporting their geroprotective effectiveness.

### Identification of neuroprotective drugs

**Fig. 6:**
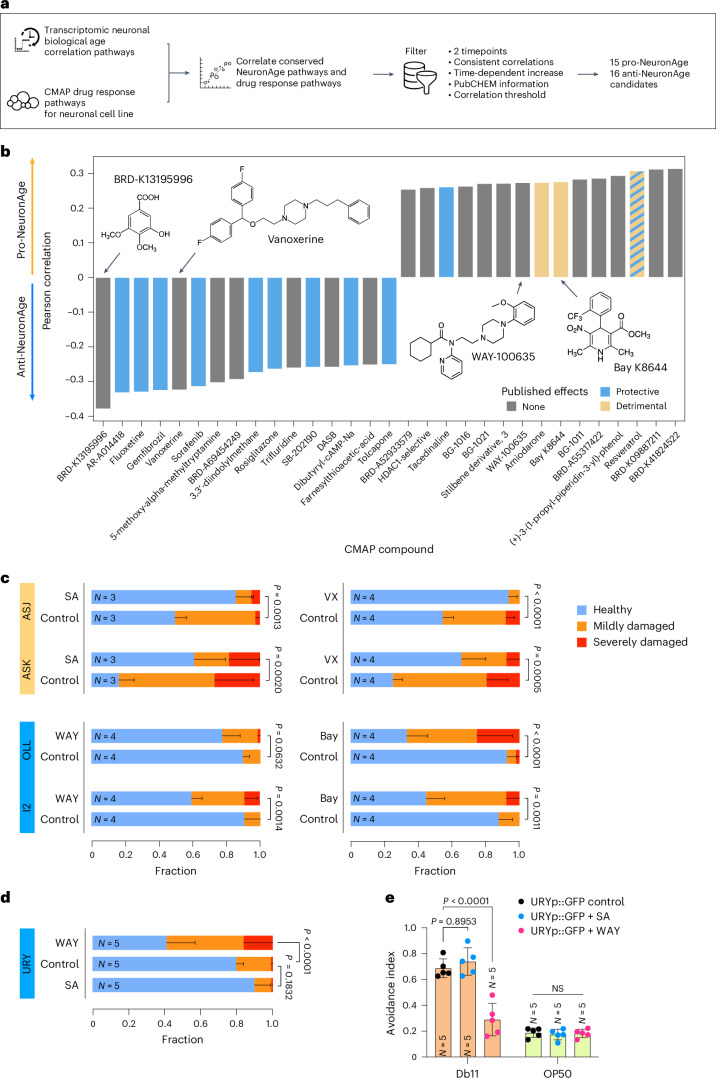
Compound prediction algorithm identifying neuroprotective/neurotoxic compounds. **a**, Flowchart depicting the in silico drug screening. We computed and correlated the conserved KEGG pathway enrichments for NeuronAge and all compounds from the CMAP dataset that are measured on the neuronal cell line NEU. To obtain a manageable list, we filtered for compounds that were measured at least twice, show consistent correlations in all measurements, have a stronger correlation at the 24-h time point compared to the 6-h time point, have information in PubCHEM and have at least an absolute correlation value of 0.25. **b**, The top anti-NeuronAge and pro-NeuronAge compounds after the filtering steps ranked according to their Pearson correlation. Previously published neuroprotective (blue) or neurotoxic (orange) compounds are indicated. Resveratrol, which has mixed evidence in the literature, is shown in both colors. Four compounds were selected for experimental validation of our predictions and are highlighted by black arrows and their structural formula is given. **c**, Fraction plots displaying the fraction of nematodes at day 1 of adulthood expressing neuronal volume markers in different neurons categorized as ‘healthy’, ‘mildly damaged’ and ‘severely damaged’ that were treated with 2.5 mM syringic acid (SA), 10 nM vanoxerine (VX), 25 nM WAY-100635 (WAY) or 250 µM (S)-(−)-Bay K8644 for 24 h. Three to four cohorts were analyzed, consisting of 10–25 individual nematodes. Data shown are the mean ± s.d. Statistical analysis was performed using a CLM with a logit link to account for the ordered categorical nature of neuronal damage scores (healthy < mild < severe). *P* values were adjusted for multiple testing using the Benjamini–Hochberg method. **d**, Fraction plot displaying the fraction size of healthy (blue), mildly damaged (orange) and severely damaged (red) URY neurons (JKM10 strain) on day 2 of adulthood after 48-h compound treatment with 25 nM WAY-100635 (WAY), 2.5 mM SA or water control. Data shown are the mean + s.d. of five independent cohorts of 10–25 nematodes each. Statistical analysis was performed using a CLM with a logit link to account for the ordered categorical nature of neuronal damage scores (healthy < mild < severe). Pairwise comparisons between conditions were performed using the ‘emmeans’ package with Tukey’s adjustment for multiple testing. **e**, Swarm plot displaying the avoidance index of compound-treated JKM10 (URYp::GFP) nematodes (25 nM WAY-100635 (WAY), 2.5 mM SA or water control for 48 h) on day 2 of adulthood. Data shown are the mean ± s.d. from five independent cohorts of 50–75 nematodes. Two-way ANOVA + Tukey post hoc test were used to test for significant differences. Source data

As the NeuronAge predictions cluster together with human neuronal chronological aging trajectories, we sought to identify small-molecule compounds that could delay neuronal aging. We used the 3,566 transcriptomes from a terminally differentiated neuronal cell line of the CMAP resource, consisting of 2,467 different molecules^13^ (Fig. 6a). Based on their correlation with the NeuronAge signature, we identified both negatively correlated (potentially neuroprotective/‘anti-NeuronAge’) and positively correlated (potentially neurotoxic/‘pro-NeuronAge’) compounds (Fig. 6b). Consistent with our experimental results, the transcriptome changes induced by CHX were inversely related to the NeuronAge signature, indicating that the beneficial effect of CHX that we observed above is mirrored in the transcriptome. After applying multiple computational filtering steps (Methods), we ranked the remaining candidate compounds by their correlation with NeuronAge (Fig. 6b). The top anti-NeuronAge hits contain several compounds (9 of 16) for which a protective effect in neurons has been previously documented, thus independently validating our approach (Fig. 6b). Two of 15 ‘pro-NeuronAge’ compounds were shown to be detrimental, while one is potentially protective, and one was described as both harmful and protective. More than half of the top hits have, however, not been tested in neurons.

In summary, 11 of 31 compounds have documented neuroprotective effects, of which 9 are predicted to revert NeuronAge, that is ‘anti-NeuronAge’, with our in silico approach. Similarly, 2 of 31 compounds are known to be neurotoxic, and both are predicted correctly to be ‘pro-aging’, giving weight to the potential that an in silico screen can identify compounds that could be repurposed as either neuroprotective or neurotoxic agents.

### Validation of neuroprotective molecule compounds

Next, we tested whether several compounds that we predicted to be anti-NeuronAge, that is, neuroprotective, could prevent the age-related functional decline of neurons. We chose two compounds, which were among the most strongly anticorrelated with NeuronAge patterns, BRD-K13195996 and vanoxerine (Fig. 6b). The chemical identity of the phenolic compound BRD-K13195996 is 3-hydroxy-4,5-dimethoxybenzoic acid, which is related to 4-hydroxy-3,5-dimethoxybenzoic acid, which is also known as syringic acid. Syringic acid is a naturally occurring secondary compound derived from edible plants and fruits, for example, olives, walnuts and grapes^30^. A correlation between the antioxidative properties of syringic acid and reduced neurotoxicity following bisphenol A insult has recently been shown^31^, yet no clear mechanism was reported so far^32^. Vanoxerine is a potent dopamine uptake inhibitor and was tested as a supportive agent in cocaine-abuse medications^33^; moreover, vanoxerine was observed to impede colorectal cancer stem cell functions by repressing G9a expression^34^. Vanoxerine is so far not reported to exhibit neuroprotective effects.

**Extended Data Fig. 6:**
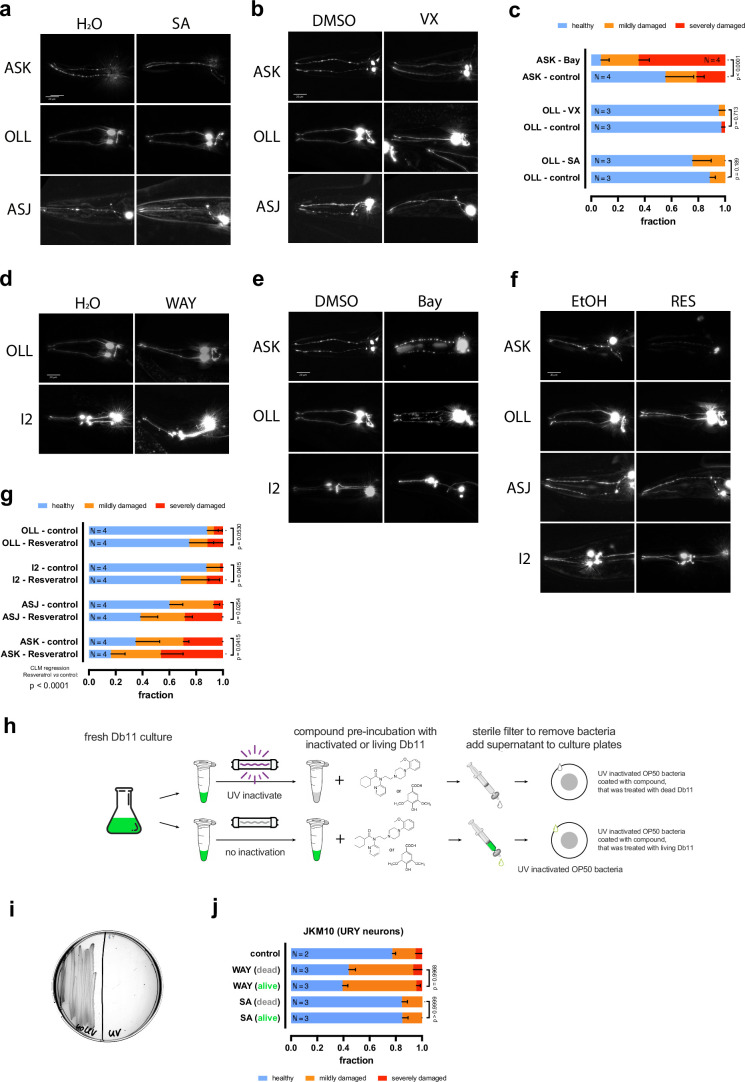
Components identified by in silico screen validation. **a**) Z-stack fluorescence images of GFP expression in ASK, OLL, ASJ neurons after 24 h treatment with water control, 2.5 mM syringic acid (SA). Maximum projections after background subtraction (Fiji – rolling ball radius 80). Scale bars indicate 20 µm. **b**) Z-stack fluorescence images of GFP expression in ASK, OLL, ASJ neurons after 24 h treatment with 10 nM vanoxerine (VX). Maximum projections after background subtraction (Fiji – rolling ball radius 80). Scale bars indicate 20 µm. **c**) Fraction-plots displaying the fraction of nematodes expressing GFP in the OLL or ASK neurons categorized as ‘healthy’, ‘mildly damaged’, and ‘severely damaged’ that were treated with 2.5 mM syringic acid (SA), 10 nM vanoxerine (VX), or 250 µM Bay K8644 for 24 h. Three to four cohorts were analysed, consisting of 10 – 25 individual nematodes each. Statistical analysis was performed using a cumulative link model with a logit link to account for the ordered categorical nature of neuronal damage scores (Healthy < Mild < Severe). Multiple test adjusted (Benjamini Hochberg) p-values are shown. **d**) Z-stack fluorescence images of GFP expression in I2 neurons after 24 h treatment with 25 nM WAY-100635 (WAY). Maximum projections after background subtraction (Fiji – rolling ball radius 80). Scale bars indicate 20 µm. **e**) Z-stack fluorescence images of GFP expression in ASK, OLL, and I2 neurons after 24 h treatment with 250 µM Bay K8644. Maximum projections after background subtraction (Fiji – rolling ball radius 80). Scale bars indicate 20 µm. **f**) Z-stack fluorescence images of GFP expression in ASK, OLL, ASJ, and I2 neurons after 24 h treatment with 1‰ ethanol, or 200 µM resveratrol. Maximum projections after background subtraction (Fiji – rolling ball radius 80). **g**) Fraction-plots displaying the fraction of nematodes expressing GFP in the OLL, I2, ASJ, or ASK neurons categorized as ‘healthy’, ‘mildly damaged’, and ‘severely damaged’ that were treated with 200 µM resveratrol for 24 h. Four cohorts were analysed, consisting of 12 – 25 individual nematodes. Statistical analysis was performed using a cumulative link model with a logit link to account for the ordered categorical nature of neuronal damage scores (Healthy < Mild < Severe). Multiple test adjusted (Benjamini Hochberg) p-values are shown. **h**) Schema of experimental strategy to test whether *Serratia marcescens* Db11 bacteria metabolise syringic acid or WAY-100635. Db11 bacteria are grown and on half is inactivated by UV-C irradiation (500 mJ/cm^2), the other half is still alive. Compounds are added separately and incubated 48 h with dead or alive bacteria. Subsequently, solution is passed trough a 22 µm syringe filter to remove bacteria. The compound containing solution is afterwards added onto a lawn of UV-C inactivated OP50 bacteria. After drying, L4 stage nematodes are added and after 24 h neurodegeneration is assessed using fluorescence microscopy. **i**) Scan of UV-C inactivation test. *Serratia marcescens* Db11 bacteria were inactivated by UV-C irradiation (500 mJ/cm^2), streaked-out onto an NGM plate, and growth was documented after 24 h. Left side shows bacteria before inactivation, right side is bacteria after inactivation. **j**) Fraction-plot displaying the fraction of nematodes expressing GFP in the URY neurons categorized as ‘healthy’, ‘mildly damaged’, and ‘severely damaged’ that were treated with 2.5 mM syringic acid (SA) or 25 nM WAY-100635 (WAY), after compounds were pretreated with living or dead Db11 bacteria for 48 h, for 24 h. Three cohorts were analysed, consisting of 10 – 25 individual nematodes. Statistical analysis was performed using a cumulative link model with a logit link to account for the ordered categorical nature of neuronal damage scores (Healthy < Mild < Severe). Pairwise comparisons between conditions were performed using estimated marginal means (emmeans) with Tukey’s adjustment for multiple testing. Source data

We applied both compounds to L4 stage nematodes for a 24-h short-term treatment. We assessed neurite degeneration in the biologically old ASJ and ASK neurons and observed a significantly reduced deterioration for both compounds (Fig. 6c and Extended Data Fig. 6a,b). In contrast, the biologically young OLL neurons, serving as controls, were not significantly affected by either of the compounds (Extended Data Fig. 6c). This indicates that both compounds interfere with the physiological degeneration process of the rapidly aging neurons and can restore a healthy neuron state.

Next, we assessed whether the NeuronAge compound predictions could also identify neurotoxic compounds and hence serve for pharmacological risk assessment. We tested the compounds WAY-100635, for which so far no adverse effects on neuron health have been reported, and Bay K8644 on the health of the young predicted I2 and OLL neurons. We observed that WAY-100635 induced significant neurite deterioration in I2 but not in OLL neurons, while Bay K8644 induced significant degeneration in both I2 and OLL neurons (Fig. 6c and Extended Data Fig. 6d,e). ASK neurons that already display neurodegeneration early in life were significantly further compromised by Bay K8644 treatment (Extended Data Fig. 6e), confirming the neurotoxic effect of Bay K8644.

Additionally, resveratrol, which was reported to have ambiguous effects, was positively correlated with NeuronAge (Fig. 6b), and we observed significantly increased neuronal damage in I2, ASJ and ASK neurons, with a similar but nonsignificant trend observed in OLL neurons (Extended Data Fig. 6f,g), supporting our predicted detrimental impact.

Taken together, we validated the anti-NeuronAge compound prediction method by identifying known neuroprotectors as well as discovering previously unknown neuroprotective molecules. In reverse, the prediction of a positive correlation with NeuronAge revealed neurotoxic compound properties.

Finally, we assessed whether drug-induced neuroprotection or degeneration preserved or compromised neuronal function, respectively. We treated URY neuron-marked animals with either WAY-100635 or syringic acid and analyzed neurodegeneration and pathogen avoidance that depends on the integrity of URY neurons (Fig. 3c). First, we confirmed that WAY-100635 exerts a significant neurotoxic effect in URY neurons (Fig. 6d). As expected from the already large healthy fraction of URY neurons, the beneficial effect of syringic acid was slight but not significant (Extended Data Fig. 6h–j).

In contrast to the drug treatment assays above, the avoidance assay necessitated the use of live bacteria. To exclude the possibility that bacterial metabolism of the compounds might confound the analysis, we pre-incubated WAY-100635 and syringic acid with either live or dead Db11 bacteria before nematode exposure (Extended Data Fig. 6h–j) and observed no significant differences thus validating this approach.

WAY-100635 treatment severely impaired pathogen avoidance from Db11 bacteria compared to solvent control treatment (Fig. 6e), while syringic acid treatment led to a small, nonsignificant increase in avoidance reaction, in line with the slight improvement in the preservation of URY neurons (Fig. 6d). This observation further corroborates the functional outcome of the morphological degeneration and substantiates that our prediction approach can be used to identify neuroprotective and neurotoxic compounds (Fig. 6a,b).

In summary, our compound validation demonstrates that predicted anti-NeuronAge compounds significantly reduce neurite degeneration and preserve neuronal function, while predicted neurotoxic compounds exacerbate neuronal damage and disturb neuronal function. These results establish our in silico screening approach as an effective approach for identifying interventions to prevent neurodegeneration, as well as for identifying potential neurotoxic agents.

## Discussion

Why distinct neuron types exhibit different susceptibilities to age-dependent degeneration and the associated neurodegenerative diseases has remained largely unclear. While differences in interindividual aging are commonly known, differential aging of organs within the same organism, and aging variance between cells of the same tissue have only recently been observed^35,36^. Using the two distinct aging clock paradigms, we found a substantial diversity of biological age predictions with specific neuron types displaying accelerated or delayed aging in chronologically young animals. Similar to such early detection of aging, epigenetic clocks in mice start advancing right after their resetting at the ground-zero stage during gastrulation^37^ and in humans they detected aging during childhood^38^. The substantial heterogeneity in neuronal aging was further supported by the age-dependent decline of long gene expression. The reliability of the age predictions was further evidenced by the corresponding age-dependent degeneration of specific neuron types. Particularly ciliated sensory neurons show an increased biological age, which may result from their exposure to the environment or could indicate that their utility is restricted to sensing optimal environmental conditions before entering reproductive age. Similarly, degeneration of the olfactory bulb, which is enriched by environmentally exposed, ciliated neurons, and loss of olfaction are among the first symptoms of Alzheimer’s disease-related degeneration in humans^39^.

The biological age-correlated transcriptional patterns associated high translational loads with accelerated aging. Age-related changes in the translational machinery have been observed across various species, and downregulation of translation has been shown to extend lifespan upon dietary restriction, downregulation of mTOR or CHX treatment^40,41^. In *C. elegans*, nucleolar size that is linked to ribosome biogenesis inversely correlates with lifespan^42^, while aging is marked by declining protein synthesis^43,44^ and increased translation errors^45^. Stochiometric changes of the ribosome lead to accumulation of protein aggregates in the brain of old *Nothobranchius furzeri*^46^, which is in line with the enrichment of ribosomal proteins in the insoluble protein fraction of old *C. elegans*^47^. Consistently, we observed that transient translation inhibition by CHX treatment is sufficient to prevent the degeneration of the fast-aging neuron types.

The transcriptional patterns of nematode neuronal biological age differences are significantly correlated with mouse and human brain chronological aging trajectories, and anticorrelated with known geroprotective interventions such as young plasma treatment or sport. This conservation shows the potential of identifying conserved mechanisms that underlie the biological aging differences and might determine the susceptibility of specific neuron types to undergo degeneration and potentially contribute to specific neurodegenerative diseases.

The conservation of transcriptome age trajectories allowed us to design an in silico compound screen using the human CMAP dataset^13^. Such approaches have previously identified geroprotective compounds that either induce a ‘youthful’ state as predicted through an age-classification approach leveraging the GTEx^48^ transcriptomic dataset^49^, by identifying compounds that counteract age-associated transcriptomic shifts using conserved aging signatures^50^, by mimicking longevity FOXO3 overexpression^51^, or induce a ‘youthful’ matreotype^52^. Comparing transcriptomic data from *C. elegans* neurons to the CMAP resource, we identified known and previously unexplored neuroprotective small-molecule compounds. Syringic acid was described to reduce oxidative stress and neuroinflammation, potentially by enhancing mitochondrial function and attenuation of acetylcholinesterase activity^53–55^. The 1,4-dialkylpiperazine derivative vanoxerine is a potent dopamine uptake inhibitor and was tested as a supportive agent in cocaine-abuse medications^33^. Vanoxerine was observed to impede colorectal cancer stem cell functions by repressing G9a expression^34^, and it was tested in clinical trials for use as an anti-arrhythmic drug^56^, but a phase III study was terminated due to the occurrence of ventricular proarrhythmia^57^. The development of analogs overcoming vanoxerine’s limitations are a work in progress^58^. By testing syringic acid and vanoxerine as examples of the top-scoring compounds, we indeed found that they extend the integrity of fast-aging neurons indicating a biological age deceleration.

Conversely, the pro-aging prediction revealed neurotoxic effects of compounds and could thus be highly valuable in risk assessment. WAY-100635 is an antagonist of the serotonin 5-HT_1A_ receptor, acting both at presynaptic autoreceptors and postsynaptic sites to modulate serotonergic signaling and increase extracellular 5-HT levels^59–61^. In addition, WAY-100635 has an affinity for the dopamine D_4_ receptor, where it acts as a potent partial agonist at higher concentrations^62,63^. The carbon-11-labeled form of WAY-100635 ([carbonyl-¹¹C]WAY-100635) is used as a radioligand for in vivo positron emission tomography imaging of 5-HT_1A_ receptors in the human brain^64–66^. While often considered safe, behavioral studies suggest context-specific effects: for example, it not only exacerbates depression-like phenotypes following mild traumatic brain injury in mice^67^, but also prevents selective serotonin reuptake inhibitor-induced sexual dysfunction when coadministered with fluoxetine^68^. WAY-100635 induced significant neurite deterioration in I2 and URY neurons, underscoring the power of our approach for predicting neurotoxicity. Bay K8644 is a potent L-type calcium channel agonist, structurally related to dihydropyridines such as nifedipine. By promoting prolonged opening of L-type voltage-gated calcium channels, Bay K8644 enhances calcium influx into neurons and has been used as a research tool to study calcium-dependent neuronal processes^69^. In animal models, Bay K8644 has been shown to induce seizures, oxidative stress and excitotoxic neuronal damage^70–72^. Consistently, we validated the predicted neurodegenerative effect of Bay K8644 across multiple neuron types. Notably, our analysis predicted resveratrol to be pro-aging, whereas the published effects have been controversial. Our in vivo validation demonstrates that resveratrol does not have a protective, but rather detrimental impact on neuronal health. Corroborating our findings, a recent study found resveratrol-induced brain atrophy in lemurs^73^. These results further validate our in silico drug screen for both identifying neuroprotective compounds that could preserve neuronal function and determining the risk of neurotoxic compounds that accelerate neurodegeneration.

Taken together, we here define the biological basis for the distinct susceptibility of neurons to undergo age-dependent degeneration. We establish the utility of using aging clocks to identify neuron-type-specific biological aging differences and based on their transcriptome profiles reveal conserved aging patterns also present in human brain aging. We show that this approach is suitable for identifying neuroprotective molecules and propose that they could be useful in delaying neuronal aging and protect from age-associated degeneration.

## Methods

### *C. elegans* culture

Nematodes were cultured on nematode growth medium (NGM) agar plates at 20 °C under standard conditions unless stated otherwise. All age statements given in this publication consider the first day of adulthood as day 1. A complete strain list can be found in the Supplementary Information.

Special care was exerted for maintaining the cultures (specifically for maintaining the strains carrying extrachromosomal arrays): To maintain good expression levels in each population, healthy-looking adults with strong, but not extreme, fluorescence were passaged. Without this careful selection, the fluorescence signals were lost over the course of a few generations.

Age synchronization for experiments was achieved by L4 picking. For all experiments, individuals displaying a medium to strong fluorescence were selected (this group consists of about 85% of the fluorescent nematodes); individuals with neither exceptionally strong fluorescence (≈3–5% of the fluorescent nematodes) nor weak fluorescence (≈5–10% of the fluorescent nematodes) were selected for experiments or passaging. The same criteria were applied when L3 larvae were selected for experiments.

### Neurite imaging

Nematodes were synchronized by L4 picking and grown for 1 day, 4 days or 7 days. For imaging, nematodes were placed in a drop of 250 mM NaN_3_ on a 2% agarose pad. Imaging was performed on a Zeiss Imager.M2 at a magnification of ×400. *z*-stacks of nematode heads/tails were acquired using 2-µm step width. Acquisition time was set between 100 ms to 3 s per plane to achieve a good signal-to-noise ratio.

### Scoring of neurite degeneration

Recorded *z*-stack images of neurons were analyzed manually, counting blebs, large spherical outgrowths, branching, breaks and necrosis on the dendrites of the analyzed neurons. Images were classified according to the degree of aberration: necrotic neurons, broken or truncated neurons, neurons with ≥10 blebs or ≥3 outgrowths were scored as ‘severely damaged’; neurons with 5–9 blebs, or 2 outgrowths were classified as ‘mildly damaged’; and neurons with <5 blebs or <2 outgrowths were classified as ‘healthy’. See Fig. 2b for exemplary images.

### Ordinal regression analysis—CLM

To statistically assess neuronal damage across conditions and time points, we used CLMs using the ordinal package in R. A logit link function was applied to estimate the cumulative probabilities of increasing damage severity. For neuron-specific analyses of damage progression over time, the time point was modeled as a numeric predictor (L4 = 0, day (D) 1 = 1, D4 = 4, D7 = 7), and separate CLMs were fit for each neuron type. Observed neuron counts per category were incorporated as weights using the weights argument. *P* values for the slope term were adjusted for multiple testing across neurons using the Benjamini–Hochberg method. To compare damage susceptibility between neuron types, a full model including neuron identity, time point and their interaction was fit (score ~ neuron × age). Estimated marginal means were computed using the emmeans package, and Tukey-adjusted *P* values were reported for all pairwise neuron contrasts. For comparisons between two conditions (for example, treatment versus control), we modeled the score as a function of the condition only (score ~ condition), again using CLMs with logit link and frequency weights. Multiple-testing correction across neurons was performed using the Benjamini–Hochberg method, where applicable.

### Compound treatment

Standard NGM plates, seeded with OP50, were inactivated with 500 mJ/cm^2^ of 251-nm UV-C light (Stratalinker 2400) and afterward were subjected to compound coating by dropwise addition of compounds directly to the agar surface. Plates were dried for at least 1 h before transferring L4-stage nematodes onto them. Nematodes were incubated with the compounds for 24 h and then used for neurite imaging. The final concentration of compounds was: CHX, 2 mM; syringic acid, 2.5 mM; vanoxerine, 10 nM; WAY-100635, 25 nM; (S)-(−)-Bay K8644, 250 µM; resveratrol, 200 µM. Control nematodes were incubated on appropriate solvent control-coated plates (either water, for syringic acid and WAY-100635, ≤5‰ DMSO, for CHX and vanoxerine, or 1‰ ethanol, for resveratrol).

### Pathogen avoidance assay

*S. marcescens* Db11 bacteria were inoculated into LB medium with kanamycin, tetracycline and streptomycin from glycerol stock and incubated overnight with shaking at 150 rpm at 25 °C. Control *E. coli* OP50 bacteria were grown in LB medium without antibiotics and incubated overnight with shaking at 150 rpm at 37 °C. NGM plates (6 cm) were cast 3 days before seeding. The plates were seeded with 350 µl bacterial solution, generating a perfect round spot in the middle of the plate and allowed to dry for 2 h. For compound treatment, 2.5 mM syringic acid or 25 nM WAY-100635 was added before seeding and allowed to dry. Around 15–30 L4-stage nematodes of strains JKM10, IG10 and MT21910 were added directly into the bacterial lawn and incubated for 48 h. Nematodes were scored for their presence on or avoidance of the lawn. Five independent cohorts of 4–5 plates each were analyzed, and the position of the nematodes was checked four times with a 5-min interval time. The aversion index was calculated as the number of nematodes outside the lawn divided by total number of nematodes. Potential compound metabolization by the living Db11 bacteria has been tested in prior experiments.

### Testing compound metabolization

For testing compounds and pathogen avoidance from *S. marcescens* Db11, bacteria could not be inactivated with UV irradiation because it would negate the aversion behavior. To test whether Db11 bacteria would metabolize the compounds, a test treatment was performed: 2.5 mM syringic acid or 25 µM WAY-100635 was incubated with living or dead Db11 bacteria for 2 days; afterward, bacteria were removed by sterile filtration and the compound-containing flowthrough was applied on UV-inactivated OP50. L4-stage JKM10 nematodes were placed on these plates, and neurodegeneration was assessed 24 h later. There was no significant difference between the compounds that were pretreated with living or dead Db11 bacteria (Extended Data Fig. 6h–j).

### Salt aversive conditioning assay

Adapted from Lim et al.^25^, nematodes were synchronized by L4 picking and grown for an additional day to their first day of adulthood, then nematodes were rinsed off the plates with M9 medium and washed twice with M9. The nematodes were split into two groups and salt-free conditioning medium or salt-containing conditioning medium (1 mM CaCl_2_, 1 mM MgCl_2_, 5 mM KPO_4_^2^^−^ pH 6.0, ±20 mM NaCl) was added. Subsequently, nematodes were incubated at 20 °C rotating at 20 rpm for 3 h.

Assay plates (3.5 cm; 2% (wt/vol) ager, 1 mM CaCl_2_, 1 mM MgSO_4_, 5 mM KPO_4_^2^^−^ pH 6.0) were cast 2 days before the experiment. To establish the NaCl gradient on the assay plates, agar plugs containing 100 mM NaCl (2% (wt/vol) agar, 100 mM NaCl, 1 mM CaCl_2_, 1 mM MgSO_4_, 5 mM KPO_4_^2^^−^ pH 6.0) were placed on one side on the plate and incubated at 23 °C for 3 h. After the gradient was established, salt-agar plugs were removed and 3 µl 500 mM NaN_3_ was added to the spot where the plug was placed. An additional 3 µl 500 mM NaN_3_ was added to the opposite site of the plate, and the plates were directly used for the assay.

After 3 h of conditioning, excessive medium was removed, and nematodes were transferred onto the center of the prepared salt-gradient assay plates. Nematodes were allowed to crawl freely on the plate according to their preferences for 30 min and at 20 °C. Afterward, nematodes that remained on the origin, that moved toward high salt and those that moved toward low salt were counted, and the chemotaxis index was calculated as: *c*_i_ = (number on high salt – number on low salt) / (total number – number on origin).

For CHX treatment, nematodes were synchronized by L4 picking and placed on an OP50-seeded NGM plate, coated with 2 mM CHX, for 24 h. After compound incubation, the nematodes were processed as described above.

### BitAge prediction

The BitAge clock^10^ was used as described previously. Briefly, each sample was binarized, that is, genes higher than the median expression value within each sample after removing genes with zero counts, were set to 1, and the remaining genes to 0. The BitAge coefficients for the 576 clock genes are added up for all genes in a given sample that is 1 after binarization. After adding the BitAge intercept, the results show the predicted biological age. For the Calico dataset, only single-neuron types were considered, that is, cells annotated as multiple neuron types were excluded. For neuron types with two biological replicates, the predicted biological ages of the replicates were averaged to obtain a single age prediction per neuron type.

### Stochastic data-based clock

The Stochastic Clock was used as described previously^12^. Briefly, each sample was log_10_-transformed after the addition of one pseudo-count. Subsequently, the samples were min–max normalized to bring each sample within the range (0, 1), and then binarized as described above. The normalized counts were then added up for all 1,010 stochastic data-based clock genes. For the Calico dataset, where some neuron types had two biological replicates, the predicted biological ages of the replicates were averaged to obtain a single age prediction per neuron type. Note that the Stochastic Clock might result in slightly different genes every time a clock is trained.

### Gene length analysis

First, we downloaded the differentially expressed genes for each neuron type and all other cells in the CeNGEN^14^ dataset (https://cengen.shinyapps.io/CengenApp/) with the statistical test ‘Wilcoxon on single cells’. This gives a list of genes with logFC, and percentage expression in the specific neuron type and all other cells. We further filtered this list of significant genes by requiring that the gene is expressed in at least 90% of cells of the specific neuron type and at most 10% in all other cell types. In total, 39 neuron types had no genes with these requirements, that is, 89 neuron types were used for further analysis. Next, we used the such-defined marker genes of the 20% oldest (57 genes) and respective 20% youngest neurons (67 genes) and calculated the density distribution of the log_10_-normalized gene lengths. To calculate a two-sided permutation test, we calculated the median log_10_ gene length of the genes and compared it to the 100,000-permutation median. The permutation for the old marker genes used 57 genes, while the permutation for the young marker genes used 67 genes of all marker genes (303) of the 89 neuron types.

In a complementary analysis, we calculated the median log_10_ gene length of the marker genes for each of the neuron types individually. These median values were correlated with the predicted biological age using a Pearson correlation and visualized with a linear regression plot.

### Definition of NeuronAge

To quantify transcriptomic aging patterns across neuronal cell types, we defined NeuronAge as the correlation between gene expression and predicted biological age with BitAge. TPM values for all 128 neuron types were obtained from the CeNGEN transcriptomic dataset, and each of the 18,475 genes was *z*-score normalized across all 128 neuron types. Predicted ages for the same neurons were derived using the BitAge clock. For every gene, we calculated the Pearson correlation between its *z*-scored TPM values and the predicted ages across all 128 neuronal cell types. The resulting correlation coefficients represent the NeuronAge transcriptional signature.

### Neuronal connectome mapping

We downloaded and adapted the Cytoscape file from Cook et al.^26^ by adding head neurons, deleting nonneuronal cell types and color coding neurons by their predicted Age with BitAge on the CeNGEN dataset.

### Median total number of synapses calculation

The NeuroType.xlsx file was downloaded from https://www.wormatlas.org/neuronalwiring.html. For each of the 128 neuron types, we summed the median total number of synapses in the head, tail and mid-body.

### Fuzzy clustering

To identify transcriptional patterns associated with the predicted neuronal age, we ranked the 128 neuron types based on their predicted age and grouped them into five bins: (1) [97, 110], (2) [110, 120], (3) [120, 130], (4) [130, 140] and (5) [140, 180]. Within each bin, we computed the median expression level for each gene. To make the genes comparable and bring them onto the same scale, we calculated the *z*-score across the five bins for each of the 9,950 genes with nonzero standard deviation. Next, we used fuzzy clustering with Mfuzz (v2.58.0)^74^ to identify transcriptional clusters across the predicted age bins. The elbow method was computed with the Dmin function of Mfuzz and indicated an optimal number of four clusters. The genes belonging to each cluster were subsequently used for a pathway enrichment analysis with clusterprofiler (v4.9.2.002)^75^, with maxGSSize = 500 and the list of all 9,950 genes as the background gene list. All significantly enriched pathways (adjusted *P* < 0.05) were identified, and redundant pathways were collapsed using semantic similarity filtering. Although this procedure substantially reduced redundancy, the remaining number of nonoverlapping pathways was still too large to display comprehensively in a single figure. For visualization purposes, we therefore selected a representative subset of pathways from each cluster based on (i) statistical significance, (ii) effect size and (iii) biological interpretability.

### Heat map

We processed several public datasets (GTEx dataset^48^, the TMS dataset^76^ and an additional mouse hypothalamus aging cohort (GSE157025), young serum injections^77^, platelet factor PF4 (ref. ^78^), sport in humans^79^, krill oil in *C. elegans*^80^) for the heat map:

1. TPM-normalized gene expression values for human brain tissues from the GTEx v8 data^81^ release (that is, amygdala, anterior cingulate cortex, caudate basal ganglia, cerebellar hemisphere, cerebellum, cortex, frontal cortex, hippocampus, hypothalamus, nuclear accumbens basal, putamen basal ganglia, substantia nigra) were correlated with the chronological age (the midpoints of the publicly available age bins).
2. The mouse aging time course for hypothalamus data from GSE157025 was downloaded and a gene-wise correlation with the chronological age calculated.
3. Whole-brain data from the TMS (GSE132040) was downloaded and edgeR (v3.40.2)^82^ was used to calculate normalized expression values. The normalized expression values were correlated to the chronological age.
4. Differentially expressed genes for mouse hippocampus data treated with the platelet factor PF4 or saline control were downloaded from GSE173254. We multiplied the logFCs by −1 to always compare treatment versus control, instead of control versus treatment.
5. Differentially expressed genes for mouse hippocampus data treated with young serum or sham were downloaded from GSE234667.
6. *C. elegans* whole-nematode data treated with krill oil from GSE207152. We used edgeR (v3.40.2)^82^ to calculate normalized expression values and calculated *z*-scores for each gene over all samples. The *z*-score-normalized expression values were used for a regression model: ${\rm{expression}}=\,{\beta }_{0}+{\beta }_{1}\times {\rm{Age}}+{\beta }_{2}\times {\rm{Krilloil}}+{\beta }_{3}\times ({\rm{Age}}\times {\rm{Krilloil}})$, where ${\beta }_{0}$ is the intercept term, ${\beta }_{1}$ is the coefficient for the Age variable, ${\beta }_{2}$ is the coefficient for the Krilloil-treatment variable, and ${\beta }_{3}$ is the coefficient for the interaction between Age and Krilloil, that is, the difference in the slope over age.
7. Differential expressed genes upon physical activity were downloaded from the supplementary data (Supplemental Table 2) from Berchtold et al.^79^.

The Pearson correlation values of all genes of (1)–(3), the logFC of all genes for (4) and (5) and the ${\beta }_{3}$ coefficients for (6) were used to calculate enriched pathways analysis with fgseaMultilevel from the fgsea R package^83^ and with nPermSimple = 1,000 for all conserved KEGG pathways between *C. elegans*, mouse and humans. For (7), the ‘anti-aging/Alzheimer’s disease’ genes were split into genes that are upregulated and, respectively, downregulated upon exercise. Both gene sets were used for an enrichment analysis with enricher from enrichplot v1.18.0 with maxGSSize = 500 and minGSSize = 5, and all genes were quantified in the supplementary data from Berchtold et al.^79^ as the background gene list. For both enrichment analyses, the enrichment fold change, that is, the number of observed genes per pathway divided by the number of expected genes per pathway, was calculated. Finally, the fold changes were combined, that is, pathways with a bigger fold-change enrichment in the downregulated genes were multiplied by −1.

The clustering was done on the normalized enrichment scores for (1)–(6) and the fold change enrichment score for (7) with the Ward method and a correlation distance matrix.

### Heat map with logFC

For the heat map with differentialy expressed genes, we processed the data in the following way:

1. CeNGENApp (https://cengen.shinyapps.io/CengenApp/) was used to find differentially expressed genes between the ten oldest predicted CeNGEN neurons (according to BitAge) and the ten youngest predicted neurons. The differentialy expressed genes were calculated with the ‘Pseudobulk:Wilcoxon’ statistical test.
2. Raw gene expression values for human brain tissues from the GTEx v8 data^81^ release (that is, amygdala, anterior cingulate cortex, caudate basal ganglia, cerebellar hemisphere, cerebellum, cortex, frontal cortex, hippocampus, hypothalamus, nuclear accumbens basal, putamen basal ganglia, substantia nigra) were normalized with edgeR and differentially expressed genes calculated between old (≥70 years) and young (≤40 years).
3. The whole-brain data from the TMS (GSE132040) were normalized with edgeR (v3.40.2)^82^, and differentially expressed genes were calculated between old (≥21 months) and young (1 month) mice.
4. The mouse aging time course data for the hypothalamus from GSE157025 were normalized with edgeR (v3.40.2)^82^, and differentially expressed genes were calculated between old (24 months) and young (5 day) mice.

The enriched pathways were computed as described above.

### CMAP

The CMAP resource uses the L1000 array, which measures 978 landmark transcripts, which can be used to infer most of the remaining transcriptome with high accuracy^13^. Here we used all available L1000 datasets for a human differentiated neuronal cell line. We downloaded the aggregated level 5 L1000 Connectivity Map^13^ data from GSE92742 and performed pathway enrichment analysis with fgseaMultilevel from the fgsea R package^83^ with nPermSimple = 1,000 and the conserved KEGG pathways between human and *C. elegans* for each of the samples in the level 5 dataset. To compare it to the NeuronAge pattern, we first calculated the *z*-score of each gene that has at least some gene counts across the 128 neurons of the CeNGEN dataset. We correlated these *z*-score-normalized genes with the biological age prediction from BitAge. The resulting Pearson correlation values for all genes were used for a pathway enrichment analysis with fgseaMultilevel and the conserved KEGG pathways between human and *C. elegans*. To identify whether any compound might revert the NeuronAge gene expression pattern on the pathway level, we correlated the normalized enrichment scores of the NeuronAge KEGG pathway enrichment analysis with all the normalized enrichment scores that we calculated from the CMAP dataset. Next, we filtered for only compounds that were tested in the neuronal cell line (‘NEU’). Compounds had to be tested at least twice, with all measurements resulting in correlations in the same direction. Additionally, we filtered for compounds that were measured at 24 h and 6 h and took only those compounds that showed a stronger correlation into the same direction at the 24-h time point compared to the 6-h time point. Lastly, we filtered out those compounds that had no information available at PubCHEM and used a correlation threshold of 0.25 (respective −0.25).

The assignment of known protective or detrimental compounds is based on literature reports: The glycogen synthase kinase 3 (GSK3) inhibitor AR-A014418 was shown to inhibit beta-amyloid-induced neurodegeneration^84^; the selective serotonin reuptake inhibitor fluoxetine protects against neurotoxicity and neurodegeneration^85^; the PPAR-alpha activator gemfibrozil exhibits neuroprotective effects via upregulating pro-survival factors and suppressing inflammation^86^; the kinase inhibitor sorafenib protects against neurodegeneration in *C. elegans*^87^; the selective aryl hydrocarbon receptor modulator 3,3’-diindolylmethane is neuroprotective and promotes brain-derived neurotrophic factor^88^; the insulin-sensitizing agent rosiglitazone exhibits neuroprotective effects in the eye and the brain^89^; the p38 MAPK inhibitor SB202190 was shown to reduce hippocampal apoptosis and rescue spatial learning as well as memory deficits in rats^90^; dibutyryl-cAMP-Na (dBcAMP) elevates cAMP levels and protects against neurodegeneration in stab wound or kainic acid injuries^91^; and the catecholamine-*O*-methyltransferase inhibitor tolcapone was shown to improve cognitive function^92^.

Bay K8644 is described to be neurotoxic^70^; and amiodarone induces neuronal apoptosis^93^ and is known to induce adverse neurological effects^94^. Tacedinaline/CI-994 is a class I histone deacetylase inhibitor, correlates positively with NeuronAge, and was shown to promote functional recovery following spinal cord injury^95^, and to enhance synaptic and structural neuroplasticity^96^. Likewise, resveratrol is potentially neuroprotective due to a hormetic response^97^, while there is evidence that it could lead to impaired brain integrity^73^.

### Correlation of neurodegeneration and GFP expression

To test whether GFP expression induces neuronal aberrations, we correlated GFP expression intensity and blebbing. In brief, fluorescence images of PY6457 (ASK neurons) and OE3010 (ASJ neurons) nematodes at the first day of adulthood were recorded using an epifluorescence microscope. PY6457 and OE3010 were selected because they carry the neuron-type-specific GFP marker as an extrachromosomal array that has a strongly varying expressivity between individuals. Blebbing was observed as the major degeneration hallmark in ASJ and ASK neurons and, therefore, used as single correlation criterion. Four cohorts of 10–20 nematodes per strain were accumulated in single plots and the correlation was analyzed by Pearson’s correlation.

### Analysis of stochasticity of neuron damage

To test if bleb appearance is fully random or linked to development of conserved structures along neurites, bleb distance from neurite start (at the site of the mouth) was measured using Fiji for every bleb on each neurite analyzed from three cohorts of 10–20 individual nematodes each. Relative positions of blebs were calculated by dividing the observed bleb’s position (for example, 55 µm) by the total length of the analyzed neurite (for example, 98 µm). Relative positions were displayed as a histogram using 12 bins, and data from three cohorts were aggregated. A Pearson correlation versus 100 random data tracks was done using GraphPad Prism 10.

To calculate 100 tracks of a random damage distribution, a Python script was used with the following assumptions: (1) a random number of blebs (in 8–20) can appear, matching observed biological variability; (2) new blebs appear primarily at random but are slightly more likely to occur near previously formed blebs, reflecting spatial clustering seen in vivo; (3) already established blebs increase chances of blebs in their vicinity; (4) blebs cannot appear at the same position; (5) the proximal 10% of the neurite corresponds to the ciliary region, where structural complexity and imaging artifacts reduce the detectability of blebs, and this was modeled by suppressing bleb placement in this region; (5) neurite lengths were randomly sampled within the empirically observed range (80–100 µm).

All simulation parameters, including neurite length distributions, bleb counts, clustering strength and the extent of the protected proximal region, were derived directly from empirical measurements of ASJ, ASK and OLL neurons imaged in vivo. For each simulated neurite, bleb positions were generated iteratively and normalized to neurite length to match the experimental measurements. The resulting 100 simulated tracks were aggregated and compared to the observed data.

### Neuron survival

Nematodes of strains JKM10 (URY neurons), OH1422 (OLL neurons), NY2067 (ASE neurons), MT21910 (I2 neurons) and PY6457 (ASK neurons) were synchronized by L4 picking. Only nematodes that showed a easily visible fluorescence under a dissecting microscope were selected and singled onto OP50-seeded NGM plates.

The presence of the neurons was scored daily. Nematodes were passaged every second day to a fresh plate. Individuals were tracked over the course of 12 days or until they died, or until all GFP-marked neurons disappeared.

Neuron loss was analyzed according to Kaplan–Meier using the accumulated data from three cohorts of each of the 12 individuals (totaling 36 nematodes per strain), and significant differences were assessed by a log-rank test.

Additionally, the first day a neuron degenerated in every single individual was scored and plotted as a histogram showing the incidence of first neuron death.

### SUnSET assay

Wild-type nematodes were synchronized by L4 picking (≈75 nematodes per cohort and condition) and placed on OP50-seeded NGM plates coated with 2 mM CHX or DMSO as control. After 24 h, plates were rinsed with S-Basal, and nematodes were collected in 1.5-ml tubes. Nematodes were washed once with S-Basal. Freshly grown OP50 bacteria were pelleted by centrifugation (20 min at 4,000 rpm at 12 °C) and resuspended in 0.1 Vol. S-Basal.

Nematodes were resuspended with 200 µl of the concentrated bacteria. Puromycin was added to a final concentration of 500 µg ml^−1^ and topped up to 1 ml with S-Basal. CHX-treated samples were supplemented with 2 mM CHX additionally. Puromycin labeling was performed for 3 h, and nematodes were rotating at 20 rpm on a spin wheel at 20 °C.

Afterward, nematodes were washed thrice with S-Basal to remove residual puromycin. Finally, nematodes were resuspended in 30 µl 4xSDS-Sample buffer, boiled at 95 °C for 5 min and then stored at −20 °C.

Frozen samples were thawed and boiled at 95 °C for 5 min. In total, 15 µl of lysate was loaded on a 12% SDS–PAGE and run at 30 mA for 2 h. Western blot transfer onto a nitrocellulose membrane was performed using Trans-blot Turbo (Bio-Rad) according to the manufacturer’s specifications (running at 2.0 A for 10 min).

Membrane was rinsed with TBS-T, Ponceau stain was applied, and imaging was performed. Ponceau staining was washed out three times with TBS-T. Membrane was blocked for 2 h in TBS-T + 5% (wt/vol) milk powder. Primary antibody incubation was done with 4G11 anti-puromycin antibody from mouse (1:1,000 dilution) in TBS-T + 2.5% (wt/vol) milk powder at 4 °C with shaking overnight. Afterward, membrane was washed three times with TBS-T and incubated with horseradish peroxidase-conjugated anti-mouse antibody (1:5,000 dilution) for 2 h. Blot was developed using ECL solution (Pierce) in an Amersham Imager 600. The acquisition time was ≈2 min.

### Fluorimetry on CHX-treated nematodes

Nematodes of strains JKM10 (URY neurons), OH1422 (OLL neurons), NY2067 (ASE neurons) and PY6457 (ASK neurons) were synchronized by L4 picking. Three cohorts per strain and condition (totaling 28–52 nematodes) were used for epifluorescence microscopy. Nematodes were prescreened to find the highest fluorescence intensity per strain; imaging settings were adjusted to not overexpose the nematode with the strongest fluorescence per strain. Imaging was performed on L4 larvae; the remaining L4 larvae from synchronization were subjected onto OP50-seeded NGM plates coated with 2 mM CHX or DMSO control and imaged after 24 h. Imaging settings were kept constant within strains. Imaging settings were kept constant between cohorts. Background fluorescence was subtracted. The mean ± s.d. was displayed as a swarm plot, and ANOVA + Tukey post hoc test were performed.

### Longitudinal CHX treatment

Three cohorts of nematodes expressing GFP in ASE, ASJ, ASK or OLL neurons have been treated with 2 mM CHX for either 24 h or 96 h starting from stage L4 onward. Neurite imaging was performed on day 4 of adulthood. Damage classification in ‘healthy’, ‘damaged’ and ‘severely damaged’ neurons was done as described in the main text. Ordinal regression (CLM) was used to test for significant differences (using Python 3.12 library: statsmodels: 0.14.3).

### Statistics and reproducibility

All data are presented as the mean ± s.d. Number of cohorts (*ℕ*), individuals (*n*) and technical replicates (*N*) are stated in the figures and their respective figure legends. The applied statistical tests are mentioned in the figure legends, and the respective *P* values are directly reported in the diagrams. All statistics are two sided unless stated otherwise. Data normality was assessed by the Shapiro–Wilk test. Independent *t*-tests were calculated with Python’s SciPy^98^ v1.5.1 stats.ttest_ind function. One-way ANOVA’s were calculated with Python’s pingouin^99^ v0.3.6 ANOVA function and the parameter ss_type = 2. Cohen’s *h*^100^ as a measure of effect size was calculated by hand with Python’s NumPy^101^ v1.18.5. Plots were generated with Python’s Seaborn^102^ v.0.11.0, matplotlib^103^ v.3.3.0 or GraphPad Prism 10. Scatterplots showing a linear regression model fit are shown with a 95% confidence interval. No statistical method was used to predetermine sample size. Sample sizes were determined based on established practices in the field and on empirical considerations. Group sizes of 10–30 animals were used for neurite degeneration scoring, and for chemotaxis assays 50–100 animals were used to balance practical feasibility and the ability to detect meaningful biological effects. No method of randomization was used to assign nematodes to experimental groups. Researchers were blinded during data analysis (folders/images were listed and assigned random alphanumeric IDs before analysis; after analysis, files were named back accordingly). If nematodes were damaged during handling, results from them were censored; whole-nematode cohorts were excluded if less than 80% of the desired animals survived until analysis day, or if nematodes were growing slowly (delayed by at least a day compared to usual growth rate of the respective strain) or were apparently sick (slow movements, begging, lethargic; this affected one cohort of MT21910 L4s in Fig. 2c and one subgroup of JKM10 in Fig. 6e).

### Reporting summary

Further information on research design is available in the Nature Portfolio Reporting Summary linked to this article.

## Supplementary information

Supplementary Information Supplementary nematode strain list.

Reporting Summary

## Source data

Source Data Fig. 1 Raw data and statistics.

Source Data Fig. 2 Raw data and statistics.

Source Data Fig. 3 Raw data and statistics.

Source Data Fig. 4 Raw data and statistics.

Source Data Fig. 5 Raw data and statistics.

Source Data Fig. 6 Raw data and statistics.

Source Data Extended Data Fig. 1 Raw data and statistics.

Source Data Extended Data Fig. 2 Raw data and statistics.

Source Data Extended Data Fig. 3 Raw data and statistics.

Source Data Extended Data Fig. 4 Raw data and statistics.

Source Data Extended Data Fig. 5 Raw data and statistics.

Source Data Extended Data Fig. 6 Raw data and statistics.

## Data Availability

The unfiltered TPM counts and the Cell Marker list was downloaded from the CENGEN dataset, assessed at https://cengen.shinyapps.io/CengenApp/. The bulk CeNGEN dataset was downloaded via https://cengen.org/storage/Barrett_et_al_2022_CeNGEN_bulk_RNAseq_data.tsv. The Calico dataset was downloaded from https://c.elegans.aging.atlas.research.calicolabs.com/data/. The neuron-specific information was assessed at https://www.wormatlas.org/. The gene length information was downloaded from https://wormbase.org/. The CMAP data were downloaded from GSE92742. Data for the heat map were downloaded from the Gene Expression Omnibus under accession numbers GSE157025, GSE132040, GSE173254, GSE234667 and GSE207152, from the supplementary data from ref. ^79^ or the GTEx v8 database via https://gtexportal.org/home/downloads/adult-gtex/bulk_tissue_expression/. Raw values and summaries of all statistics used are included in the Extended Data. Source data are provided with this paper. All data supporting the findings of the study are also available from the corresponding author upon request.

## Code availability

All scripts and code used are available on GitHub via https://github.com/Meyer-DH/NeuronAging/.
