# Supplementary material for: Aging clocks delineate neuron types vulnerable or resilient to neurodegeneration and identify neuroprotective interventions
Source: Nat Aging. 2026 Feb 3;6(4):849–68. doi: 10.1038/s43587-026-01067-5 (PMC13099438; doi:10.1038/s43587-026-01067-5)
Supplement: Supplementary file 1 — Supplementary nematode strain list. [file 43587_2026_1067_MOESM1_ESM.pdf]

nature aging

Supplementary information https://doi.org/10.1038/s43587-026-01067-5

Aging clocks delineate neuron types vulnerable or resilient to neurodegeneration and identify neuroprotective interventions

In the format provided by the authors and unedited

Supplementary Information

Table of Content

- nematode strain list

# – nematode strain list –

Strain name	Genotype	Neuronal Expression	Origin
N2	wild type	--	CGC
OH1422	*otIs138* [*ser-2p3::GFP + rol-6(su1006)*] X	OLL	Hobert Lab via CGC
PY6457	Ex1[*srbc-64p1::gfp + unc-122::dsRed*]	ASK	Sengupta Lab
CX3596	*kyIs128* [*str-3::GFP + lin-15(+)*]; *lin-15B&lin-15A(n765)* X.	ASI	Bargmann Lab via CGC
MT21910	*nEx2065* [*gur-3prom::GFP + lin-15(+)*]; *lin-15AB(n765ts)* X.	head neurons, I2	Horvitz Lab via CGC
OE3010	*ofEx4* [*trx-1::GFP + lin-15(+)*]; *lin-15B&lin-15A(n765)* X	ASJ	Swoboda Lab via CGC
BL5717	*inIs179* [*ida-1p::GFP*] II.; *him-8(e1489)* IV.	subset, PHC	Blumenthal Lab via CGC
JKM10	*jksIs10* [*tol-1p::GFP*]	URY	Kirstein Lab
NY2067	*ynIs67* [*flp-6p::GFP*] III.; *him-5(e1490)* V.	ASE	Li Lab via CGC
CB928	*unc-31*	pan neuronal	CGC
