# Supplementary material for: Aging clocks delineate neuron types vulnerable or resilient to neurodegeneration and identify neuroprotective interventions
Source: Nat Aging. 2026 Feb 3;6(4):849–68. doi: 10.1038/s43587-026-01067-5 (PMC13099438; doi:10.1038/s43587-026-01067-5)
Supplement: Supplementary file 2 — Reporting Summary [file 43587_2026_1067_MOESM2_ESM.pdf]

nature portfolio

Corresponding author(s): Christian Gallrein, David H. Meyer, Björn Schumacher

Last updated by author(s): Dec 15, 2025

Reporting Summary

Nature Portfolio wishes to improve the reproducibility of the work that we publish. This form provides structure for consistency and transparency in reporting. For further information on Nature Portfolio policies, see our Editorial Policies and the Editorial Policy Checklist.

Statistics

For all statistical analyses, confirm that the following items are present in the figure legend, table legend, main text, or Methods section.

n/a	Confirmed	
☐	☒	The exact sample size (*n*) for each experimental group/condition, given as a discrete number and unit of measurement
☐	☒	A statement on whether measurements were taken from distinct samples or whether the same sample was measured repeatedly
☐	☒	The statistical test(s) used AND whether they are one- or two-sided<br>*Only common tests should be described solely by name; describe more complex techniques in the Methods section.*
☐	☒	A description of all covariates tested
☐	☒	A description of any assumptions or corrections, such as tests of normality and adjustment for multiple comparisons
☐	☒	A full description of the statistical parameters including central tendency (e.g. means) or other basic estimates (e.g. regression coefficient) AND variation (e.g. standard deviation) or associated estimates of uncertainty (e.g. confidence intervals)
☐	☒	For null hypothesis testing, the test statistic (e.g. *F*, *t*, *r*) with confidence intervals, effect sizes, degrees of freedom and *P* value noted<br>*Give P values as exact values whenever suitable.*
☒	☐	For Bayesian analysis, information on the choice of priors and Markov chain Monte Carlo settings
☐	☒	For hierarchical and complex designs, identification of the appropriate level for tests and full reporting of outcomes
☐	☒	Estimates of effect sizes (e.g. Cohen's *d*, Pearson's *r*), indicating how they were calculated

Our web collection on statistics for biologists contains articles on many of the points above.

Software and code

Policy information about availability of computer code

Data collection	*Provide a description of all commercial, open source and custom code used to collect the data in this study, specifying the version used OR state that no software was used.*
Data analysis	Code for BitAge and Stochasitic aging clocks, Fuzzy clustering, CMAP and compound-in-silico-screen, neurite damage stochasticity simulation, and further data analysis and visualization are provided via public Github repository: https://github.com/Meyer-DH/NeuronAging<br>The following packages were used: python v3.6.10, scipy v1.5.1, pingouin v0.3.6,numpy v1.18.5, pandas v.1.1.5, statsmodels v.0.11.1, seaborn v0.11.0, matplotlib v3.3.0, GraphPad Prism 10, R v4.2.2, edgeR v3.40.2, fgsea v1.24.0, enrichplot v1.18.0, clusterprofiler v4.9.2.992, Mfuzz v2.58.0

For manuscripts utilizing custom algorithms or software that are central to the research but not yet described in published literature, software must be made available to editors and reviewers. We strongly encourage code deposition in a community repository (e.g. GitHub). See the Nature Portfolio guidelines for submitting code & software for further information.

Data

Policy information about availability of data

All manuscripts must include a data availability statement. This statement should provide the following information, where applicable:

- Accession codes, unique identifiers, or web links for publicly available datasets
- A description of any restrictions on data availability
- For clinical datasets or third party data, please ensure that the statement adheres to our policy

Further information and requests for resources and reagents should be directed to and will be fulfilled by the corresponding author, Björn Schumacher (bjoern.schumacher@uni-koeln.de). Public dataset accession codes are reported in Figure Legends and Material And Methods, and are listed in the supplement.
The unfiltered TPM counts and the Cell Marker list was downloaded from the CENGEN dataset, assessed at https://cengen.shinyapps.io/CengenApp/ .
The bulk CeNGEN dataset was downloaded via https://cengen.org/storage/Barrett_et_al_2022_CeNGEN_bulk_RNAseq_data.tsv .
The Calico dataset was downloaded from https://c.elegans.aging.atlas.research.calicolabs.com/data .
The neuron-specific information was assessed at https://www.wormatlas.org/ .
The gene length information was downloaded from https://wormbase.org/ .
The CMAP data were downloaded from GSE92742.
Data for the heatmap were downloaded either from the GEO database: GSE157025, GSE132040, GSE173254, GSE234667, GSE207152. From the Supplementary data from PMID: 30927700. Or the GTEx v8 database: https://gtexportal.org/home/downloads/adult-gtex/bulk_tissue_expression
Raw values and summaries of all employed statistics are included in the 'SourceData' file in the Extended Data.

Research involving human participants, their data, or biological material

Policy information about studies with human participants or human data. See also policy information about sex, gender (identity/presentation), and sexual orientation and race, ethnicity and racism.

Reporting on sex and gender	does not apply
Reporting on race, ethnicity, or other socially relevant groupings	does not apply
Population characteristics	does not apply
Recruitment	does not apply
Ethics oversight	does not apply

Note that full information on the approval of the study protocol must also be provided in the manuscript.

Field-specific reporting

Please select the one below that is the best fit for your research. If you are not sure, read the appropriate sections before making your selection.

☒ Life sciences ☐ Behavioural & social sciences ☐ Ecological, evolutionary & environmental sciences

For a reference copy of the document with all sections, see nature.com/documents/nr-reporting-summary-flat.pdf

Life sciences study design

All studies must disclose on these points even when the disclosure is negative.

Sample size	Sample sizes were determined based on established practices in the field and on empirical considerations. Group sizes of 10 - 30 animals were employed for neurite degeneration scoring and for chemotaxis assays 50 - 100 animals were employed to balance practical feasibility and the ability to detect meaningful biological effects.
Data exclusions	If nematodes were damaged during handling, results from them were censored; whole nematode cohorts were excluded if less than 80% of the desired animals survived until analysis day, or if nematodes were growing slowly (delayed by at least a day compared to usual growth rate of the respective strain), or were apparently sick (slow movements, begging, lethargic) [this affected one cohort of MT21910 L4s in Fig.2c; and one sub-group of JKM10 in Fig.6e].
Replication	For every experiment at least three independent cohorts of at least 10 animals were analysed.
Randomization	No method of randomization was used to assign nematodes to experimental groups
Blinding	Prior to data analysis folders/files/images were listed and assigned random alphanumeric IDs before analysis; after analysis files were named back accordingly.).

Reporting for specific materials, systems and methods

We require information from authors about some types of materials, experimental systems and methods used in many studies. Here, indicate whether each material, system or method listed is relevant to your study. If you are not sure if a list item applies to your research, read the appropriate section before selecting a response.

Materials & experimental systems

n/a	Involved in the study
☐	☒ Antibodies
☒	☐ Eukaryotic cell lines
☒	☐ Palaeontology and archaeology
☐	☒ Animals and other organisms
☒	☐ Clinical data
☒	☐ Dual use research of concern
☒	☐ Plants

Methods

n/a	Involved in the study
☒	☐ ChIP-seq
☒	☐ Flow cytometry
☒	☐ MRI-based neuroimaging

Antibodies

Antibodies used	mouse–anti-Puromycin (monoclonal, 4G11, Sigma-Aldrich: MABE342)<br>anti-mouse-HRP (Sigma-Aldrich: AP308P)
Validation	The antibody detects ectopic puromycin incorporation into nascent amino acid chains; in-experiment validation was done by using a control group that was not exposed to puromycin, yielding no signal in westernblot at all.

Animals and other research organisms

Policy information about studies involving animals; ARRIVE guidelines recommended for reporting animal research, and Sex and Gender in Research

Laboratory animals	Transgenic C. elegans strains were used.
Wild animals	no wild animals were employed
Reporting on sex	Only hermaphrodite nematodes were used.
Field-collected samples	no field collection has been done
Ethics oversight	does not apply

Note that full information on the approval of the study protocol must also be provided in the manuscript.

Plants

Seed stocks	--
Novel plant genotypes	--
Authentication	--
